# Pan-cancer analysis of genomic scar patterns caused by homologous repair deficiency (HRD)

**DOI:** 10.1038/s41698-022-00276-6

**Published:** 2022-06-09

**Authors:** E. Rempel, K. Kluck, S. Beck, I. Ourailidis, D. Kazdal, O. Neumann, A. L. Volckmar, M. Kirchner, H. Goldschmid, N. Pfarr, W. Weichert, D. Hübschmann, S. Fröhling, C. Sutter, C. P. Schaaf, P. Schirmacher, V. Endris, A. Stenzinger, J. Budczies

**Affiliations:** 1 Institute of Pathology, Heidelberg University Hospital, 69120 Heidelberg, Germany; 2 Center for Personalized Medicine (ZPM) Heidelberg, 69120 Heidelberg, Germany; 3 German Center for Lung Research (DZL), Heidelberg site, 69120 Heidelberg, Germany; 4 Institute of Pathology, TUM School of Medicine, Technical University of Munich, 81675 Munich, Germany; 5 German Cancer Consortium (DKTK), 69120 Heidelberg, Germany; 6 Division of Translational Medical Oncology, NCT Heidelberg and DKFZ, 69120 Heidelberg, Germany; 7 NCT Molecular Diagnostics Program, NCT Heidelberg and DKFZ, 69120 Heidelberg, Germany; 8 Institute of Human Genetics, Heidelberg University Hospital, 69120 Heidelberg, Germany

**Keywords:** Predictive markers, Ovarian cancer

## Abstract

Homologous repair deficiency (HRD) is present in many cancer types at variable prevalence and can indicate response to platinum-based chemotherapy and PARP inhibition. We developed a tumor classification system based on the loss of function of genes in the homologous recombination repair (HRR) pathway. To this end, somatic and germline alterations in *BRCA1/2* and 140 other HRR genes were included and assessed for the impact on gene function. Additionally, information on the allelic hit type and on *BRCA1* promoter hypermethylation was included. The HRDsum score including LOH, LST, and TAI was calculated for 8847 tumors of the TCGA cohort starting from genotyping data and for the subcohort of ovarian cancer also starting from WES data. Pan-cancer, deleterious *BRCA1/2* alterations were detected in 4% of the tumors, while 18% of the tumors were HRD-positive (HRDsum ≥ 42). Across 33 cancer types, both *BRCA1/2* alterations and HRD-positivity were most prevalent in ovarian cancer (20% and 69%). Pan-cancer, tumors with biallelic deleterious alterations in *BRCA1/2* were separated strongly from tumors without relevant alterations (AUC = 0.89), while separation for tumors with monoallelic deleterious *BRCA1/2* alterations was weak (AUC = 0.53). Tumors with biallelic deleterious alterations in other HHR genes were separated moderately from tumors without relevant alterations (AUC = 0.63), while separation for tumors with such monoallelic alterations was weaker (AUC = 0.57). In ovarian cancer, HRDsum scores calculated from WES data correlated strongly with HRDsum scores calculated from genotyping data (*R* = 0.87) and were slightly (4%) higher. We comprehensively analyzed HRD scores and their association with mutations in HRR genes in common cancer types. Our study identifies important parameters influencing HRD measurement and argues for an integration of HRDsum score with specific mutational profiles.

## Introduction

Defective DNA repair is as a hallmark of cancer^1,2^. One core DNA repair mechanism fixing double stranded DNA breaks and interstrand cross-links is homologous recombination repair (HRR). Defective HRR, termed homologous recombination deficiency (HRD), is observed in several common cancer types including ovarian, breast, pancreatic and prostate cancer and renders tumors more sensitive to platinum-based chemotherapies as well as to PARP inhibition via synthetic lethality^3,4^. HRD is caused by aberrations in genes encoding the HRR pathway, such as *BRCA1, BRCA2*, *ATM, ATR, BRIP1, PALB2*, *RAD51B/C/D*, and others, and may lead to genomic instability and characteristic patterns of genomic scars, a phenomenon that can be diagnostically utilized. In this context, *BRCA1* and *BRCA2* represent the most studies genes showing impaired gene function by germline mutations, somatic mutations or epigenetic modifications^5–12^. The role of aberrations in other HRR genes and their association with genomic instability is less well understood^4^. Diagnostic HRD testing can focus on the interrogation of likely deleterious/deleterious mutations in HRR genes^13^ or on evidence for genomic instability. Several signatures of genomic instability associated with the HRD phenotype have been identified, including loss of heterozygosity (LOH)^14^, telomeric allelic imbalance (TAI)^15^, and large-scale transitions (LST)^16^. Recent clinical research has demonstrated their predictive potential by evaluating patients’ response to platinum-based therapies and PARP inhibitors in the context of breast and ovarian cancer^3,17^. Using TCGA datasets, we comprehensively analyzed major parameters influencing HRD detection and the association of genomic instability with aberrations in HRR genes within and across common cancer types including ovarian cancer. Our study defines important cornerstones of diagnostic HRD testing and contribute to a better understanding of complex biomarkers.

## Results

We performed a pan-cancer and tumor type specific analysis of HRD-induced genomic scar patterns in a total of 8847 tumors from the TCGA project with whole exome sequencing (WES) and genotyping data available. The study covered 33 cancer types which are referred to by acronyms (Supplementary Table 1).

### Mutation status of *BRCA1/2* and other HRR pathway genes

**Fig. 1:**
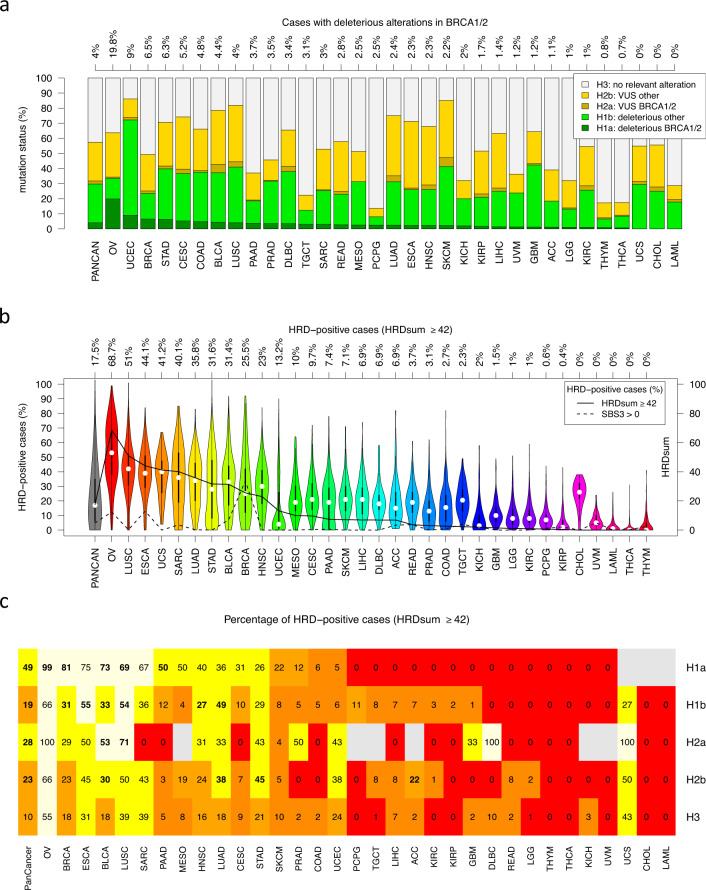
Pan-cancer analysis of HRR gene mutations and genomic scar signatures (TCGA cohort). **a** Tumors with deleterious alterations in *BRAC1/2* (H1a), in other HHR genes (H1b), VUS in *BRCA1/2* (H2a) and in other HRR genes (H2b). **b** Distribution of the HRDsum score and percentage of cases classified as HRD-positive by HRDsum (cutpoint: 42) and by SBS3 (cutpoint: 0). HRDsum was calculated starting from genotyping data (SNP arrays). **c** Percentage of HRD-positive (HRDsum ≥ 42) tumors in the five classes H1a-H3 (grey = no cases). Significant enrichments of HRD-positive tumors compared to H3 are shown in bold face.

Germline and somatic alterations in *BRCA1/2* were the primary criterion, alterations in other HRR genes were the secondary criterion for tumor classification with respect to HRR status (Supplementary Fig. 1). As potentially deleterious alteratiuons, substitutions, indels and homozygous deletions were considered. For the second criterion, 140 HRR genes from the list in Lord and Ashworth^18^ including *ATM*, *ATR*, *BAP1*, *BRIP1*, *FANCA/C/D2/E/F*, *PALB2*, and *RAD51B/C/D* were analyzed. As a result, we obtained a 5-tier classification scheme separating tumors with deleterious *BRCA1/2* alterations (class H1a, 4% of all tumors), tumors with deleterious alterations in other HRR genes (class H1b, 26%), tumors with VUS in *BRCA1/2* (class H2a, 2%), tumors VUS in other HRR genes (class H2b, 26%) and tumors not affected by relevant genetic alterations (class H3, 43%). The percentage of tumors in class H1a varied strongly between cancer types, OV: 20% (by far the highest percentage), UCEC: 9%, BRCA: 7%, STAD: 6%, CESC and COAD: about 5%, BLCA, LUSC, PAAD, and PRAD: about 4%, and less than 3.5% in the other cancer types (Fig. 1a).

### Genomic scar signatures

The level of HRDsum varied markedly within and between cancer types (Fig. 1b). Using the cutpoint HRDsum ≥42, a total of 1552 tumors (17.5%) were HRD-positive and the following ten cancer types included the highest percentage of HRD-positive tumors: OV (68.7%), LUSC (51%), ESCA (44.1%), UCS (41.2%), SARC (40.1%), LUAD (35.8%), STAD (31.6%), BLCA (31.4%), BRCA (25.5%), and HNSCC (23%). Five of these cancer types (OV, LUSC, STAD, BLCA, and BRCA) overlapped with top ten cancer types in the list ordered by the frequency of *BRCA1/2* alterations.

In addition to HRDsum, the levels of specific mutational signatures (single base substitution signatures SBS3 and SBS8) and the number of deletions with microhomology (included in the indel signatures ID6 and ID8) have been suggested as alternative markers for HRD^19,20^. In most cancer types, less HRD-positive tumors where identified by SBS3 > 0 than by HRDsum ≥ 42. By contrast, in BRCA, 301 (34%) tumors were SBS3-positive, while 228 (26%) tumors were HRDsum-positive and 135 (15%) were both. Using WES data compared to WGS data is associated with a lower sensitivity for detection of SBS and ID signatures: In the study cohort, only 31 (0.4%) of the tumors had SBS8 > 0 and only 340 (3.8%) of the tumors had TIB ≥ 50, the threshold setting used by Nguyen et al.^20^ for the HRD-classification based on WGS data. However, the median TIB was 4 and 3, respectively, in OV and BRCA showing that HRD-classification based on deletions with microhomology is not feasible using WES data, but requires WGS data.

We calculated the percentage of HRD-positive (HRDsum ≥ 42) cases in the mutation classes H1a-H3 (Fig. 1c). In the pan-cancer analysis, significantly more tumors were HRD-positive in the classes H1a, H1b, H2a, and H2b (49%, 19%, 28% and 23%) compared to class H3 (10%). In OV, BRCA, BLCA, LUSC, and PAAD, significantly more tumors were HRD-positive in class H1a (99%, 81%, 73%, 69% and 50%) compared to class H3 (55%, 18%, 18%, 39% and 5%). In ESCA, LUSC, LUAD, BLCA, BRCA, and HNSC, significantly more tumors were HRD-positive in class H1b (55%, 54%, 49%, 33%, 31%, and 27%) compared to class H3 (31%, 39%, 18%, 18%, 18%, and 16%). In LUSC, and BLCA, significantly more tumors were HRD-positive in class H2a (71%, and 53%) compared to class H3 (39%, and 18%). In STAD, LUAD, BLCA, and ACC, significantly more tumors were HRD-positive in class H2b (45%, 38%, 30%, and 22%) compared to class H3 (21%, 18%, 18%, and 2%).

### Relation of HRD with TMB, mutational signatures and dMMR

We analyzed the correlation of TMB, TIB and the SBS mutational signatures with HRDsum (Supplementary Fig. 2). In about half of the cancer types (16/33) we detected positive correlations between TMB and HRDsum, including PAAD, BRCA, OV, and PRAD (*R* = 0.6, 0.52, 0.43, and 0.43). Simultaneously with positive correlations between TMB and HRDsum we often observed positive correlations between TIB and HRDsum as well as between specific mutational signatures and HRDsum. These included the clock-like signatures SBS1 and SBS5, the APOBEC-related mutational signatures SBS2 and SBS13, as well as SBS3 which correlated significantly with HRDsum in BRCA, OV, and BLCA (*R* = 0.4, 0.26, and 0.17). These observations are consistent with a scenario in which HRR-defective tumors simultaneously accumulate genomic scars contributing to HRDsum and mutations according specific mutational signatures, TMB, and TIB.

In UCEC, COAD, and STAD we detected negative correlations of TMB, TIB, and specific mutational signatures with HRDsum. These included the clock-like signatures SBS1 and SBS5, the dMMR-related signatures SBS15, SBS20, and SBS44, as well as the *POLE*-mutation-related signatures SBS10a and SBS10b. In line with these negative correlations, HRDsum was significantly lower in microsatellite unstable or deleterious *POLE/D1*-mutated tumors compared to tumors without these alterations (Supplementary Fig. 3). Only 4 (1.2%) of the tumors showing these alterations compared to 175 (21.5%) tumors without these alterations had HRDsum ≥ 42. These results are consistent with the theory that—while defects in single DNA repair systems are a hallmark of cancer^2^—simultaneous defects in more than one DNA repair system are unfavorable for cancer cell viability. Here, we observed mutual exclisivity of defects affecting single strand repair (dMMR) and defects affecting double strand repair (HRD).

### Association of *BRCA1/2* status and genomic scar signatures

**Fig. 2:**
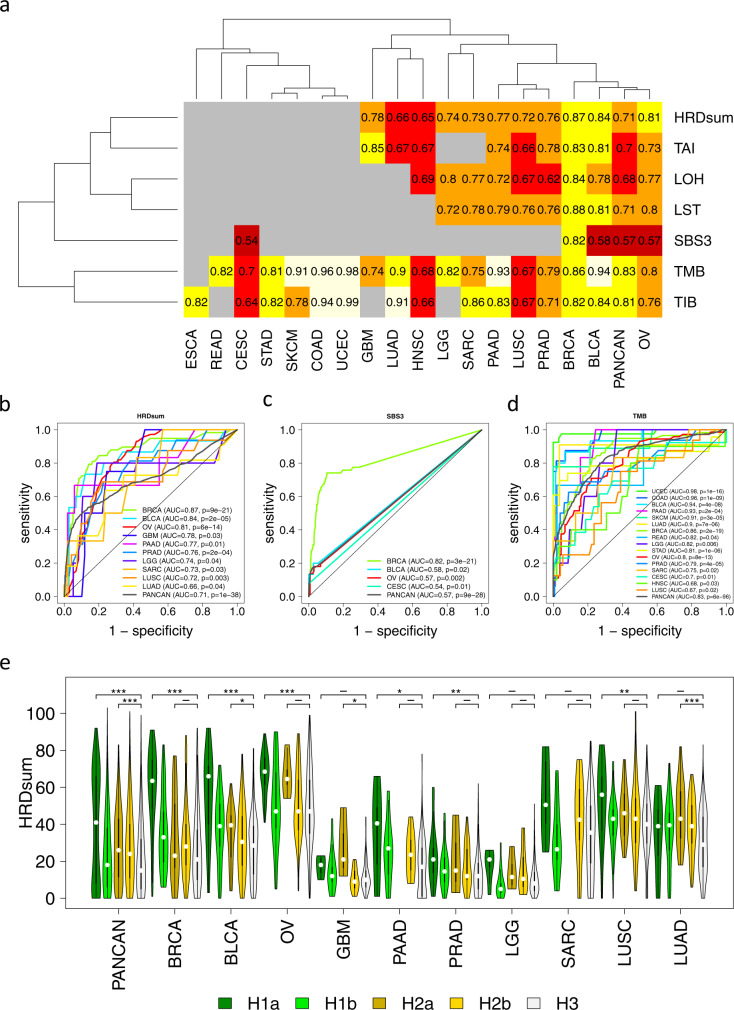
Predictivity of genomic scar signatures for deleterious *BRCA1/2* alterations (H1a vs. H3). **a** ROC analysis of seven genomic signatures. Significant positive associations are shown in heat colors (AUC > 0.5, FDR = 10%). Non-significant or negative associations are shown in grey. **b**–**d** ROC curves for the cancer types showing significant positive association with HRDsum, SBS3, and TMB. **e** Significantly enhanced levels of HRDsum in tumors with deleterious *BRCA1/2* mutations (H1a vs. H3) in BRCA, BLCA, OV, SARC, and PRAD. In the pan-cancer analysis, HRDsum levels were significantly enhanced in classes H1a, H1b, H2a and H2b compared to H3. *P < 0.05, **P < 0.01, and ***P < 0.005.

HRDsum, TAI, LST, LOH, TMB, TIB, and SBS3 were analyzed for the power to distinguish between deleteriously *BRCA1/2*-altered tumors and tumors without relevant alterations in HRR genes (H1a vs. H3, Fig. 2). By HRDsum, significant separation was achieved pan-cancer and within the eleven cancer types BRCA, BLCA, PAAD, OV, PRAD, GBM, LUSC, SARC, LGG, HNSC, and LUAD. Pan-caner, an AUC = 0.71 was reached, while AUCs were greater than 0.8 for three of the listed cancer types. By SBS3, significant separation was achieved pan-cancer and within BRCA, BLCA, OV, and CESC (AUC = 0.82, 0.58, 0.57, and 0.54). By TMB, significant separation was achieved pan-cancer and within the seventeen cancer types UCEC, COAD, PAAD, BLCA, SKCM, LUAD, BRCA, LGG, READ, STAD, OV, PRAD, SARC, GBM, HNSC, CESC, and LUSC (AUC = 0.98 to 0.67). Among these, UCEC, COAD and STAD were known to include dMMR and *POLE/D1*-mutated tumors and SKCM and LUAD were known to harbor high TMB connected with exposure to ultra-violet light and tobacco smoke, respectively. It is possible that parts of the correlations between TMB levels and *BRCA1/2* alterations are induced by monoallelic (MA) *BRCA1/2* mutations that act as passengers and do not cause HRD. To test this hypothesis, we separated *BRCA1/2* and other HRR gene alterations with respect to the hit type.

### Biallelic versus monoallelic HRR gene mutations

**Fig. 3:**
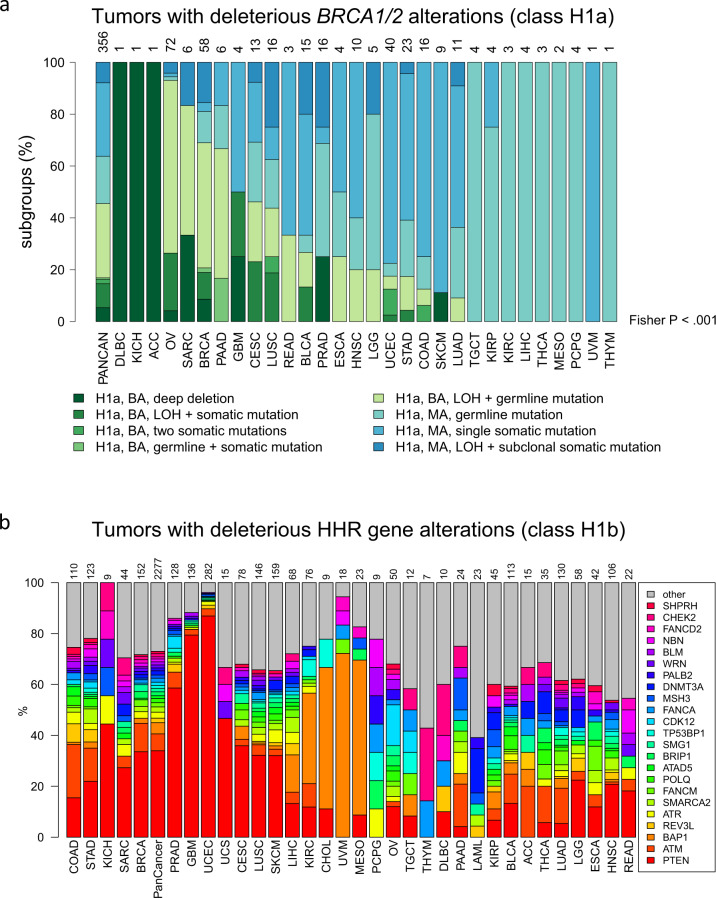
In-depth analysis of the classes H1a (tumors with deleterious *BRCA1/2* alterations) and H1b (tumors with deleterious alterations in other HRR genes). **a** Splitting of the deleterious *BRCA1/2* alterations with respect to mutation type and the number of affected alleles. BA biallelic alteration, MA monoallelic alteration. **b** Splitting of the HHR gene alterations with respect to the affected gene.

Separation of biallelic (BA) and MA hits represents an important subclassification of mutations in tumor suppressor genes, as the former hit type is associated with loss of function, while the latter one is associated with retained function according to Knudson’s second hit hypothesis^21^. Applying the approach to separate between BA and MA alterations developed in the Methods section, we found that the percentage of BA alterations of all *BRCA1/2* alterations varied across cancer types (Fig. 3a). While a high percentage of BA alterations was detected in OV, SARC, BRCA, and PAAD (93%, 83%, 69%, and 67%), it was lower in CESC, LUSC, BLCA, and PRAD (46%, 44%, 27%, and 25%) and very low in UCEC, STAD, COAD, SKCM, and LUAD (18%, 17%, 13%, 11%, and 9%).

Next, we separately analyzed tumors with class H1a, BA and tumors with class H1a, MA alterations and the power of HRDsum to separate these from class H3 tumors (Supplementary Fig. 4). Restriction to BA alterations considerably improved the classification results, especially for BLCA, PAAD, BRCA and PRAD with a close to perfect separation (AUC = 0.98, 0.97, 0.95 and 0.94). Vice versa, restriction to MA alterations worsened the classification results and a significant separation was only obtained for BLCA, BRCA, PRAD, and LUSC. Together, these results support the validity of the second hit hypothesis in context of HRD and thus underline the importance to include the hit type in the classification of *BRCA1/2* alterations.

### HRR pathway genes beyond *BRCA1/2*

The genes that were most frequently affected by deleterious alterations varied across the cancer types (Fig. 3b). *PTEN*, *ATM*, and *BAP1* were the most frequently affected genes pan-cancer (34%, 7%, and 4% of all H1b cases). Furthermore, *PTEN* was the most frequently affected gene in BRCA and PRAD (34% and 59%), while *CDK12* was the most frequently affected gene in OV (16%) and *ATM* was the most often affected gene in PAAD (7%).

For 142 HRR genes including *BRCA1/2*, we analyzed the association of the level of HRDsum with mutation status pan-cancer and in each of the cancer types (Supplementary Fig. 5). Pan-cancer, significantly higher HRDsum scores were detected in tumors with alterations in 24 genes including *BRCA1, BRCA2, RAD51B*, and *RAD51C*. Within specific cancer types, significantly higher HRDsum scores were detected in *BRCA1*-mutated OV and BRCA; in *BRCA2*-mutated OV, BRCA, PRAD, BLCA, and GBM; in *PTEN*-mutated OV and PRAD; in *ATM*-mutated THCA and LUAD; in *BAP1*-mutated KIRC and KIRP and in others.

### *BRCA1* promotor hypermethylation

**Fig. 4:**
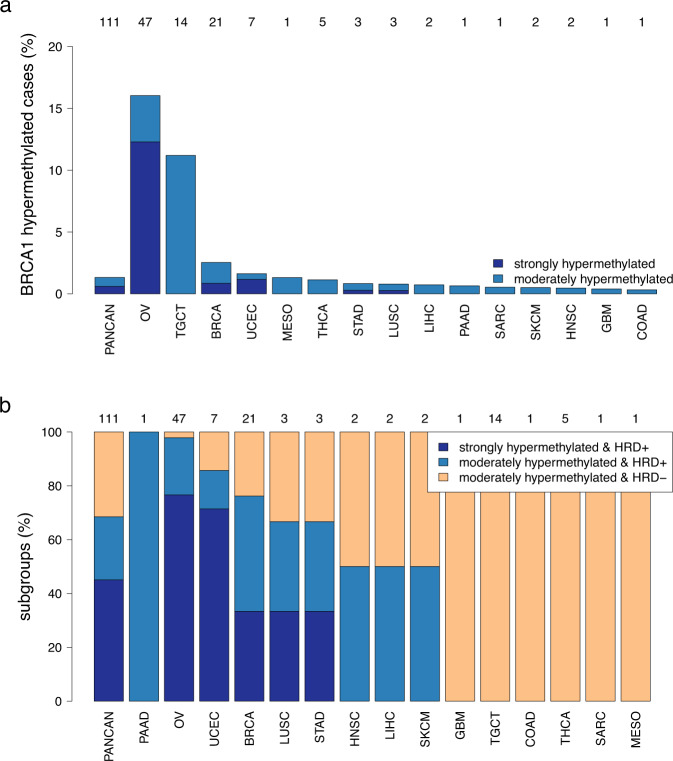
Analysis of *BRCA1* promotor-hypermethylation. **a** Percentages of strongly and moderately hypermethylated tumors pan-cancer and in specific cancer types. Top line: number of methylated tumors of each cancer type. **b** Association of methylation status with HRD-positivity (HRDsum ≥ 42). All strongly hypermethylated tumors and 43% of the moderately hypermethylated tumors were HRD-positive.

The relevant CpG sites and thresholds for the detection of *BRCA1* hypermethylation were determined in correlation analyses of beta-values and gene expression levels (Supplementary Fig. 6). Pan-cancer, we detected 111 (1.3%) *BRCA1* hypermethylated cases, whereof 50 were strongly hypermethylated and 61 were moderately hypermethylated (Fig. 4a). The largest proportions of *BRCA1*-hypermethylated cases were detected in OV (16%), TGTC (11%), BRCA (2.5%) and UCEC (1.6%). *BRCA1*-hypermethylation was associated with high HRDsum (Fig. 4b, Supplementary Fig. 7): All of the strongly *BRCA1*-hypermethylated tumors were positive for HRD (HRDsum ≥ 42), while 43% of the moderately *BRCA1*-hypermethylated tumors were HRD-positive. The latter proportion was much higher when restricting to OV, for which all excluding a single exception (98%) of the moderately *BRCA1*-hypermethylated tumors were HRD-positive.

### Integration of HRR gene mutations, hit type and *BRCA1* methylation

**Fig. 5:**
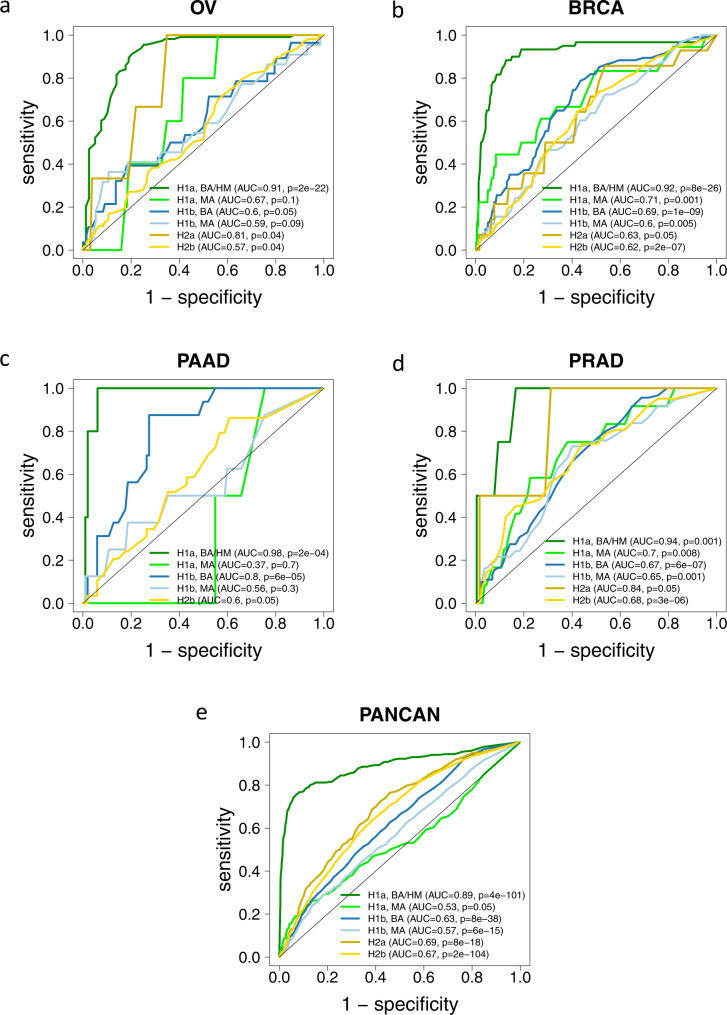
Separation of tumors with alterations in HRR genes (classes H1a-H2b) from tumors without such alterations (class H3) by HRDsum. Tumors with *BRCA1* promotor-hypermethylation (HM) were included in class H1a, BA (biallelic alteration). **a** Ovarian cancer (OV). **b** Breast cancer (BRCA). **c** Pancreatic adenocarcinoma (PAAD). **d** Prostate adenocarcinoma (PRAD). **e** Across 33 cancer types (PANCAN).

We split mutation classes H1a and H1b by hit type (BA vs. MA mutations) and formed a combined class “H1a, BA/HM” by combing the tumors with deleterious, BA *BRCA1/2* alterations with the *BRCA1*-hypermethylated tumors. The separation of the six resulting classes from H3 by HRDsum was investigated using ROC curves (Fig. 5).

Pan-cancer, all six other classes could be significantly separated from class H3. The by far best separation was obtained for tumors with BA deleterious *BRCA1/2* alterations (class H1a, BA/HM; AUC = 0.89), while separation of tumors with BA deleterious mutations in other HRR genes was inferior (class H1b, BA; AUC = 0.63) and not better than the separation of tumors with VUS in BRCA1/2 (class H2a, AUC = 0.69) and VUS in other HRR genes (class H2b, AUC = 0.67). For separation, the hit type (BA or MA) did matter strongly for class H1a mutations and less strongly for class H1b mutations.

In OV, BRCA, PAAD, and PRAD the class “H1a, BA/HM” showed the by far strongest and most significant separation from H3 (all AUC ≥ 0.9). Out of the other H1 classes, all three classes “H1a, MA”, “H1b, BA” and “H1b, MA” were significantly separated in BRCA and PRAD, while only “H1b, BA” was significantly separated in OV and PAAD. These results underline that the classification of HHR gene alterations by can be improved the inclusion of the hit type and BRCA1 hypermethylation.

We repeated the analyses for the H-classification based on a more concise gene list of 20 genes involved in HRR instead of the 140 genes included so far (Supplementary Figs. 8, 9). Using the short gene list, 72.7% of the tumors were assigned to class H3 without any relevant alteration, considerably more than 42.6% of the tumors that were assigned to this class using the long gene list. By contrast, class H1b, MA was separated from H3 in a similar way (AUC = 0.64 and AUC = 0.63), while class H2b separated from H3 slightly better for the long gene list (AUC = 0.61 and AUC = 0.67). Thus, the strength of separation did not improve by shortening of the gene list supporting the view that there are genes involved in HRD beyond the 20 genes in the short list.

Optimal cutpoints for HRDsum were determined by maximizing Youden’s index for the separation of class H1a BA/HM from class H3 (Supplementary Table 2). In the analysis across cancer types an optimal cutpoint of 43 was obtained. In the analysis of the nine cancer types with significant separation of the two classes, cutpoints were higher in BLCA, OV, LUSC, and BRCA (66, 54, 49 and 45) compared to SARC, HNSC, STAD, PAAD, and PRAD (39, 39, 37, 37, and 21). Leave-one-out cross-validation (loocv) resulted in higher Youdens’s indices for the optimal cutpoint compared to the cutpoint of 42 for BRCA, OV, STAD, PRAD and SARC.

### Explanations for high HRD scores

Out of the 1552 tumors with HRDsum ≥ 42 in the pan-cancer analysis, 9% and 3% had BA and MA deleterious *BRCA1/2* alterations (class H1a), 14% and 13% had BA and MA deleterious alterations in other HRR genes (class H1b) and 5% were *BRCA1*-hypermethylated (Supplementary Fig. 10). Furthermore, 3% and 31% had VUS in *BRCA1/2* (class H2a) or other HRR genes (class H2b), while a large remainder of 23% of tumors did not have any detected genetic or epigenetic alteration associated with HRD (class H3). Considering the 250 ovarian carcinoma with HRDsum ≥ 42, larger proportions of 27% and 18% had BA deleterious *BRCA1/2* alterations and *BRCA1*-hypermethylated promoters, while the proportion of tumors in the other mutation classes was lower. The percentage of the remaining tumors without relevant alterations (22%) was similar to the pan-cancer analysis.

To uncover further genetic causes of high HRD scores, we analyzed the association of HRDsum ≥ 42 with mutations in the subcohort of class H3 tumors. The analysis was carried cancer type specific and for each cancer type all mutations with a prevalence of at least 5% were included. For *TP53*, we found a significantly higher mutation rate of 68% compared to 24% for the tumors with high HRD scores compared to the tumors with low HRD scores after summary over the 33 cancer types (*p* = 1.7E−50). Analyzing specific cancer types, *TP53* was significantly (*p* < 0.05) associated with high HRD scores in BLCA, BRCA, ESCA, OV, PRAD, STAD and UCEC. Summarizing the significances across cancer types and taking into consideration multiple testing, mutations of no other genes were associated with high HRD scores of H3 tumors. In summary, a high percentage of tumors with HRDsum ≥ 42 had TP53 mutations for both for the subcohort of class H3 tumors (68%) and for the entire cohort (75%).

### Whole exome sequencing for the extraction of HRD scores

**Fig. 6:**
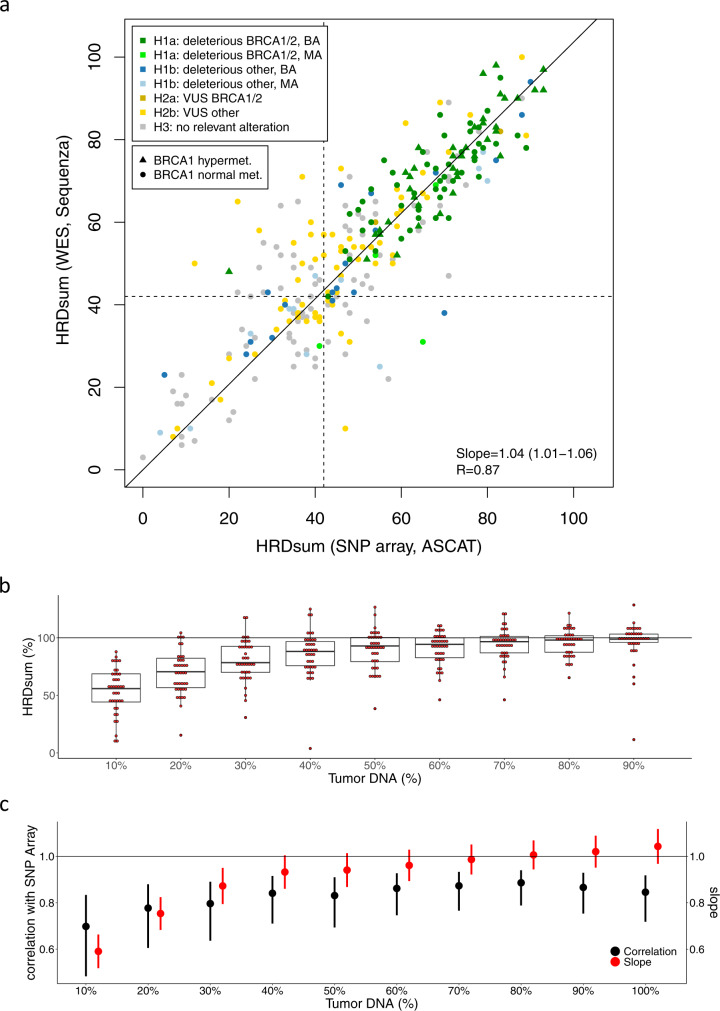
Validation of HRDsum scores calculated from WES for the TCGA ovarian carcinoma cohort. **a** Correlation of HRDsum between WES and SNP array data. Each tumor is colored according to the HRR gene mutation class H1-H3. Linear fit with intercept fixed at zero. *R* = Pearson correlation. **b/c** Analysis of the influence of tumor purity on determination of HRDsum by WES. In silico dilution series including 10%, …, 100% reads from the tumor DNA samples and 90%, …, 0% of the reads from the normal DNA samples. **b** HRDsum scores of the diluted samples expressed as percentages of the corresponding undiluted samples. Box-plot elements show the 5%, 25%, 50%, 75% and 95% quantile of the distribution of HRD scores. **c** Comparison between HRDsum scores of WES and SNP arrays by Pearson correlations and by fitting linear models without intercepts fixed at zero. Pearson correlations and slopes are shown including 95% confidence intervals.

Starting from the WES data of paired tumor and normal samples, allele-specific copy numbers and subsequently TAI, LST, LOH, and HRDsum were calculated in the ovarian cancer subcohort (TCGA-OV). A strong and highly significant correlation (*R* = 0.87) was observed between HRDsum scores calculated from WES data and the ones calculated from SNP array data (Fig. 6a). WES data resulted in slightly higher (4%) HRDsum scores compared to SNP array data.

We analyzed the capacity of HRDsum to separate between ovarian cacinomas showing genetic and epigenetic alterations related to HRD from tumors not showing these alterations (Supplementary Table 3). Based on WES data, using the cutpoint 42, sensitivity and specificity to separate tumors of class “H1a BA/HM” from tumors not in this class were 100% and 40.2%. Using a higher cutpoint 50, sensitivity and specificity changed to 97.8% and 54.4%. PPV was 43.3% when using cutpoint 42 and 49.5% when using cutpoint 50. Results obtained based on the genotyping data were similar.

Finally, we analyzed the influence of tumor purity on the determination of HRDsum by WES. In the TCGA project, tumor samples were preselected for high tumor purity and most of the tumor samples included had tumor purity of at least 60% or even 80% (Supplementary Fig. 11). In a correlation analysis of HRDsum and tumor purity, we detected significant correlations only for two of 21 cancer types (BRCA: *R* = 0.11, *p* = 0.0015; PRAD: *R* = 0.15, *p* = 0.0017). Using linear model, we estimated a downshift of HRDsum of 1.8 and of 1.4 per 10% tumor purity for breast cancer and for prostate cancer.

To systematically analyze the effect on tumor purity on HRDsum, we generated an in silico dilution series including 10%, …, up to 100% of the reads of tumors DNA samples and 90%, …, down to 0% of the reads of the corresponding normal DNA samples for 40 ovarian carcinomas (Supplementary Figs. 12, 13). For each of the diluted samples, the HRDsum score was calculated and displayed as percentage of the corresponding undiluted sample (Fig. 6b). We observed a systematic downshift of the HRDsum with increasing dilution that was moderate for dilutions up to 40%. We also compared the HRDsum scores from the dilution series to the corresponding scores derived from the SNP array data of the undiluted samples (Fig. 6c). For dilutions including at least 20% of tumor DNA, HRDsum correlated strongly with the result from genotyping (*R* ≥ 0.8). However, only for dilutions up to 40%, slopes obtained from linear regression were close to one, but considerably lower than one for stronger dilutions. The real-world TCGA data as well as out in silico dilution analysis support the view that a close to unbiased determination of HRDsum using WES is feasible for samples with tumor purity of 40% or more, while lower purity is connected with a systematic downshift of the HRD scores.

## Discussion

Diagnostic detection of HRD in tumor samples will become increasingly important in the next few years. HRD may be measured by causal deleterious mutations in HRR genes, as performed in a recent trial for prostate cancer patients^13^, or by the consequence of such events resulting in genomic instability. Recently, HRD was approved as a predictive biomarker that identifies ovarian cancer patients benefiting from combinatorial treatment with bevacizumab and olaparib^17^. In this context, HRD was defined as a multiparametric genomic instability biomarker interrogating LOH, LST, and TAI (HRDsum) and measured by a commercially available assay.

Here, we analyzed the association between genetic and epigenetic alterations in components of the HRR pathway and the clinically approved HRDsum score. Furthermore, we investigated several parameters influencing HRD detection. HRDsum was most prevalent in ovarian cancer followed by squamous cell carcinoma of the lung, esophageal carcinoma and uterine carcinoma. HSRsum correlated positively with TMB and negatively with microsatellite instability. The percentage of tumors with high HRDsum was much lower in tumors with other HRR gene alterations compared to tumors harboring deleterious/likely deleterious *BRCA1/2* alterations. In line with genetic considerations^21,22^, our data also show that classification as BA or MA hit also influences the probability of association with high HRD scores. Notably, alterations in *BRCA1/2* as well as in 140 other HHR genes including were associated with higher HRDsum scores. Significantly enhanced HRDsum scores were detected or deleterious alterations in those genes, but also for alterations presently annotated as VUS. Hypermethylated *BRCA1* was associated with high HRDsum and most abundant in ovarian cancer (16%) and testicular germ cell tumors (11%). Collectively, these data contribute to a better understanding of mixed results in current clinical studies analyzing *BRCA1/2* and sets of non-*BRCA1/2* HRR genes.

The data presented here show that optimal cutpoints of HRDsum are tumor-type dependent and that a cutpoint of 42 - for the first time introduced in a study on triple-negative breast cancer^9^ and employed in recent ovarian cancer trials^23,24^ - may result in suboptimal patient classification in other cancer types. This should be considered in future clinical trial designs and is in line with data by Davies et al.^19^ who also noted different scores of their HRDdetect tool when used outside its original detection scope (breast cancer). Pan-cancer, 23% of the tumors (22% for ovarian cancer) with HRDsum ≥ 42 belonged to the class H3 where no genetic or epigenetic explanation is available in line with data presented by Davies et al.^19^. In line with Knijnenburg et al.^1^, we found that *TP53* were associated with higher HRD scores. Specifically, 75% of all tumors with HRDsum ≥ 42 and 68% of the H3-tumors with HRDsum ≥ 42 were *TP53*-mutated. A recent study on *BRCA1/2* alterations reported restriction of the selective pressure for biallelic inactivation, zygosity-dependent phenotype penetration and sensitivity to PARP inhibition to cancer types associated with increased heritable cancer risk in *BRCA1/2* carriers^25^. These results advocate to combine HRD scores (such as HRDsum) and the analysis of alterations in specific genes of the HRR pathway (for example using the H-classification system developed in this study) for a sensitive and specific detection of HRD across cancer types. Our comparative analysis of a 140- and a 20-gene list suggests usage of a broad gene inclusion criterium at the present time compatible with the presently incomplete knowledge on the mechanistic causes of HRD.

With the excepotion of SBS3 in breast cancer, mutational signatures^26^ derived from WES data were not suited to support the detection of HRD. A more accurate determination of mutational signatures and better support of HRD detection can be achieved by WGS instead of WES^19,20^. We found a high correlation between HRDsum scores calculated from genotyping datasets (SNP array) and WES implying that both technologies can be employed for HRD detection when focusing on HRD sum. WES additionally offers the opportunity to measure single genes and other genomic biomarkers (e.g. TMB^27^) in a single assay. In silico dilution experiments showed that HRDsum scores are stable for tumor purity of 40% or more, while lower tumor purity can influence HRDsum. A systematic downshift of HRD scores was introduced by lower tumor purity which has clinical implications for clinical samples of low tumor purity and a HRDsum score below or close to the threshold of 42. Thus, tumor purity needs to be carefully controlled in the clinical implementation of HRD analysis.

Our results complement and add to pioneering work by Davies et al.^19^ who developed a multiparametric model, termed HRDdetect, primarily for the detection of HR-deficient breast cancer using whole genome sequencing data. While their approach utilizes several mutational signatures identified and conceptually developed by Alexandrov et al.^26^ to provide an integrated view on the consequential genomic scar arising from an unknown functionally abrogating event in an HRR gene, we aimed at identifying parameters influencing HRDsum, a biomarker which is now used in routine clinical practice, by looking at likely causal mutational patterns shaping and influencing HRD sum scores in several major cancer types. In line with the notion of Davies et al.^19^, our clinic-centered work shows that a multilayered approach, which reflects causes of HRD including the functional diversity of the HRR pathway—in our case by integrating specific mutational classes of HRR genes as well as biallelic vs. monoallelic status—and consequences (e.g. HRDsum) is superior to a monoparametric identifier solely using HRDsum. The relevance of looking closer at the specific causes of HRD is also implicitly reflected by data from the Profound trial^13^ showing that non-BRCA HRR genes investigated in this trial are not equally associated with significant clinical response to PARP inhibitors in prostate cancer. According to our data, an integrated analysis approach would likely have led to improved biology-driven selection of patients that benefit from treatment. As we have shown this would necessitate a different testing strategy that includes parallel testing of tumor and germline DNA as well as coverage of a broader genomic footprint.

A limitation of the study is the of use publicly available molecular data, which do not allow to analyze wet-lab parameters that may influence HRD results. Furthermore, for the pan-cancer TCGA cohort analyzed here, WES but not WGS data were available precluding a more accurate determination of mutational signatures, quantification of the deletions with microhomology as well as comprehensive downsampling experiments to compare WGS, WES, and gene panels. Another limitation was the limited number of cases with HRD for cancer types with low HRD prevalence. The highest numbers of *BRCA1/2*-defective tumors (class H1a BA/HM) were available for OV (*n* = 113), BRCA (*n* = 60), UCEC (*n* = 14), TGCT (*n* = 14) and LUSC (*n* = 10), while less than ten BRCA1/2-defective tumors were at hand for all other cancer types. For cancer types with low HRD prevalence, HRD scores should be further studied in larger cohort or cohorts enriched for HR-deficient tumors.

Our study contributes to uncovering the genetic and epigenetic underpinnings of HRD. For detection of HRD in routine diagnostics, we suggest to combine the HRDsum score, defined as sum of LOH, TAI, and LST, and the mutational status of specific HRR genes, thereby integrating analysis of cause and of consequence of HRD. Our proposed classification of HRR genes and variant types supports the systematic evaluation of alterations in HRR genes. In line with Kundson’s two hit hypothesis and the strong dependence of HRD scores on the allelic hit type observed in this study, biallelic but not monoallelic alterations should be considered primarily for the therapeutic exploitation of HRD. The comprehensive analysis of HRD scores in 33 cancer types suggests that cutpoints for HRDsum should be defined in a tumor-type specific manner. A one-size fits all approach is unlikely to work according to our analysis. Further studies are warranted to fix the cutpoints in cancer types with low HRD incidence. Our study indicates that determining HRDsum by WES of paired tumor and normal samples is feasible and advocate to control for tumor purity as a critical confounding factor of HRDsum measurement. Digital image analysis might contribute to a more accurate determination of tumor cell content. Collectively, our results support the implementation of HRD testing in a clinical setting and have implications for the design of future clinical trials.

## Methods

### Study cohort

The study cohort included tumors of 33 cancer types that were molecularly characterized in the TCGA project. All analyzes were based on the data generated in the TCGA project. Germline alteration calls were obtained from Huang et al.^28^. Somatic alteration calls were obtained from the PanCanAtlas web page^29,30^. For each tumor, tumor mutational burden (TMB) was calculated as the total number of missense mutations and tumor indel burden (TIB) was calculated as the total number of small insertion and deletions (indels). Methylation data (beta-values) and gene expression data prepared as described in Hoadley et al.^31^ were obtained from the PanCanAtlas web page^29^. Single base mutational signatures (SBS) extracted by SigProfiler^32^ were obtained from the Synapse portal^33^. Allele-specific copy numbers estimated using the ASCAT algorithm and SNP array data as input were obtained from the GDC Data Portal^34^. Aligned WES data (BAM files) of ovarian cancer and paired normal samples (TCGA-OV cohort) were obtained from the GDC Data Portal^34^ (data access approved by NIH, project #15058). Estimates of allele-specific copy numbers from WES data of paired tumor and normal samples were calculated using Sequenza^35^. A total of 8847 tumors for which mutation data, allele-specific copy numbers und SBS signature estimates were available, were included in the study.

### Variant classification

Germline and somatic variants in *BRCA1/2* and 140 other HRR genes (including *ATM*, *ATR*, *BAP1*, *BLM*, *BRIP1*, *CDK12*, *CHEK1*, *CHEK2*, *FANCA*, *FANCC*, *FANCD2*, *FANCE*, *FANCF*, *MRE11*, *NBN*, *PALB2*, *RAD51B*, *RAD51C, RAD51D* and *WRN*) listed in the Supplementary Material of Lord and Ashworth^18^ were classified using a 5-tier classification system. As results, variants were annotated as either “deleterious”, “likely deleterious”, “variant of unknown significance (VUS)”, “likely polymorphism” or “polymorphism”. As an alternative approach, we used the more concise list of 20 genes (genes listed above) from the main text of Lord and Ashworth.

In detail, germline variants were classified as “deleterious” or “likely deleterious” when listed as “pathogenic” or “likely pathogenic” in Huang et al. ^28^. Homozygous deletions were called from the ASCAT calls of copy number alterations, whenever the copy number calls of both alleles were zero. In this case the alteration was classified as “deleterious”. Somatic *BRCA1/2* mutations were classified according to the database of the ENIGMA consortium^36^. In case the variant could not be found in this database, we used a combination of the following five databases for classification: ClinVar^37^, the University of Utah Department of Pathology and ARUP Laboratories mutation database^38,39^, the Breast Cancer Information Core (BIC) database^40^, Leiden Open Variation Database (LOVD)^41^ and our in-house ionLIMS database. Variants were classified as “VUS” case of conflicting assessments by the databases. Somatic mutations in other HRR genes were classified according to the annotations in ClinVar.

Variants, that could not be found in any of the above six databases were classified as follows: Frame shift, nonsense, or splice site mutations were classified as “likely deleterious”. Intronic or synonymous mutations were classified as “polymorphisms”. All other variants were classified as “VUS”.

### Tumor classification according to HHR gene variants

Integrating information on germline and somatic mutations of *BRCA1*, *BRCA2* and the 140 other HRR pathway genes we classified the TCGA tumors according to the following 5-tier classification scheme (Supplementary Fig. 1):

- H1a: (likely) deleterious alteration in *BRCA1/2*
- H1b: (likely) deleterious alteration in another HRR gene
- H2a: VUS in *BRCA1/2*
- H2b: VUS in another HRR gene
- H3: wildtype status or alteration with retained function for all HRR genes.

Primarily, the classification depended on mutations of *BRCA1/2*: In case of a deleterious mutation, likely deleterious mutation or a homozygous deletion in *BRCA1/2* tumors were assigned to class H1a. Secondarily, mutations in the other HRR genes were taken into consideration: In case of a deleterious mutation, likely deleterious mutation or a homozygous deletion in one of these genes, tumors were assigned to class H1b. Thirdly, in case of VUS in *BRCA1/2* or in one of the other HRR genes, tumors were assigned to classes H2a or H2b. Finally, the remaining tumors that did not harbor relevant alterations in any of the 142 HRR genes were assigned to class H3.

### Biallelic versus monoallelic alterations

According to the second hit hypothesis^21^, tumor suppressor genes require inactivation of both alleles to drive cancer. Thus, dependent if a single allele or both alleles were affected, we classified hit type of a mutation as either biallelic (BA) or monoallelic (MA). From the ASCAT data, we extracted the total copy number CN = A + B as well as the copy number of the major and minor alleles, A and B. Assuming that at least half of the reads of the corresponding alleles included the mutations, we used the thresholds α = A/(2 × CN) and β = B/(2 × CN) for the variant allele frequency (VAF). Prior to comparison with the thresholds, VAFs were corrected for tumor purity by dividing the original VAFs by the histopathological determined tumor cell content. Alterations were classified as BA if one of the following criteria was fulfilled:

1. Homozygous deletion, i.e. CN = 0
2. LOH and germline mutation
3. LOH and deleterious somatic mutation with VAF ≥ α
4. Germline and deleterious somatic mutation with VAF ≥ β
5. Two deleterious somatic mutations (VAF1 ≥ VAF2) with VAF1 ≥ α and VAF2 ≥ β

Alterations classified as MA if none of the above criteria was fulfilled.

### *BRCA1* methylation

Methylation levels of four out of eight interrogated CpG sites in the *BRCA1* promotor (cg04658354, cg08993267, cg10893007 and cg19531713) showed strong negative correlation with *BRCA1* mRNA expression (Supplementary Fig. 6). Thus, *BRCA1* methylation status was determined based on these four indicative sites. Tumors were classified as strongly *BRCA1*-hypermethylated, if at least one of the CpG sites had beta ≥ 0.6 (*N* = 50, 0.6%). Tumors were classified as moderately *BRCA1*-hypermethylated, if at least one of the CpG sites had beta ≥ 0.2, but none of them had beta ≥ 0.6 (*N* = 61, 0.7%). All other tumors were classified as *BRCA1*-unmethylated.

### Calculation of HRD scores

For the entire TCGA cohort, allele-specific copy numbers were estimated from SNP array data using the ASCAT algorithm^42^. The ASCAT estimates were downloaded from the GDC Data Portal^29,34^. For the ovarian cancer subcohort (TCGA-OV) allele-specific copy numbers were additionally estimated from WES data using Sequenza^35^. To this end, BAM files of paired tumor and normal samples of TCGA-OV were downloaded from the GDC Data Portal^34^. The HRD scores TAI^15^, LST^16^, LOH^14^, and HRDsum^9^ (= TAI + LST + LOH) were calculated from allele-specific copy numbers using HRDscar^43^.

### In silico dilution experiment

Tumor purity was high in TCGA-OV (median: 90%, 95% CI 83% - 99%, Supplementary Fig. 11). To simulate ovarian cancer samples with a lower tumor purity, we replaced 10%, 20%, …, 90% of the reads in the tumor DNA BAM file by randomly drawn reads of the corresponding normal DNA BAM file. In case there were not enough reads in the normal DNA BAM file, we doubled each read prior to the random draw.

### Statistical analysis

Correlations between genomic signatures were analyzed using Spearman’s rho and two-sided testing for statistical significance. Association of the levels of genomic signatures with *BRCA1/2* mutation status was analyzed using receiver operator characteristic (ROC) curves, areas under the ROC curves (AUC) and assessed for significance using the one-sided Wilcoxon test. ROC curves and AUC were calculated using the R-package ROCR^44^. AUCs of different ROC curves were compared using the DeLong test^45^. Optimized cutpoints for the genomic signatures in each of the cancer types were calculated by maximizing Youden’s index^46^. Fold changes of HRDsum between HHR gene mutated and unmutated tumors were assessed for statistical significance using the one-sided Wilcoxon test.

Heatmaps were the preferred way to present results for a multiplicity of genomic signatures in a multiplicity of cancer types. For control of the false discovery rate (FDR), *p*-values corresponding to the displayed statistics were corrected for multiple testing using the Benjamini-Hochberg method^47^ including both heatmap dimensions (signatures × cancer types). Only significant results (FDR < 10%) were displayed as boxes in heat colors. Heatmaps were generated using the function heatmap.2 in the R package gplots^48^. Hierarchical clustering was performed using the Euclidean distance as dissimilarity measure and the average linkage method to calculate the distance between clusters.

The association of mutations and HRDsum ≥ 42 was analyzed separately in each of the 33 cancer types and assessed using Fisher’s exact test. Only 1200 genes that were mutated in at least 5% of the 8847 samples were included in the analysis. The *p*-values were combined to pan-cancer p-vales using Fishers method^49^. *P*-values for the genes included in the analyses were corrected using the Benjamini-Hochberg method and the FDR was controlled at 10%.

### Reporting summary

Further information on research design is available in the Nature Research Reporting Summary linked to this article.

## Supplementary information

Supplementary Material

REPORTING SUMMARY

## Data Availability

The data analyzed in this study were generated in the TCGA project, were published before and are available via the GDC Data Portal of the National Cancer Institute.

## Code availability

The R code for the statistical analyses and for figure generation is available upon request from the authors.
