# Supplementary material for: Pan-cancer analysis of genomic scar patterns caused by homologous repair deficiency (HRD)
Source: NPJ Precis Oncol. 2022 Jun 9;6:36. doi: 10.1038/s41698-022-00276-6 (PMC9184602; doi:10.1038/s41698-022-00276-6)
Supplement: Supplementary file 1 — Supplementary Material [file 41698_2022_276_MOESM1_ESM.pdf]

Abbreviation	Cancer Type
ACC	Adrenocortical carcinoma
BLCA	Bladder Urothelial Carcinoma
BRCA	Breast invasive carcinoma
CESC	Cervical squamous cell carcinoma and endocervical adenocarcinoma
CHOL	Cholangiocarcinoma
COAD	Colon adenocarcinoma
DLBC	Lymphoid Neoplasm Diffuse Large B-cell Lymphoma
ESCA	Esophageal carcinoma
GBM	Glioblastoma multiforme
HNSC	Head and Neck squamous cell carcinoma
KICH	Kidney Chromophobe
LAML	Acute Myeloid Leukemia
LGG	Brain Lower Grade Glioma
LUSC	Lung squamous cell carcinoma
MESO	Mesothelioma
OV	Ovarian serous cystadenocarcinoma
PAAD	Pancreatic adenocarcinoma
PCPG	Pheochromocytoma and Paraganglioma
PRAD	Prostate adenocarcinoma
READ	Rectum adenocarcinoma
SARC	Sarcoma
SKCM	Skin Cutaneous Melanoma
STAD	Stomach adenocarcinoma
TGCT	Testicular Germ Cell Tumors
THCA	Thyroid carcinoma
THYM	Thymoma
UCEC	Uterine Corpus Endometrial Carcinoma
UCS	Uterine Carcinosarcoma
UVM	Uveal Melanoma

Supplementary Table 1: The 33 cancer types included in the study.

cancer type	n (H1a BA or HM)	n (H3)	AUC	p	p (corrected)	optimal cutpoint	J (cutpoint = 42)	J (optimal cutpoint)	J (loocv)
**PANCAN**	271	2961	0.89	1.3E-99	3.4E-98	43	0.67	0.68	0.67
**BRCA**	60	445	0.92	4.4E-26	5.7E-25	45	0.73	0.75	0.74
**OV**	113	113	0.88	6.1E-23	5.3E-22	54	0.52	0.65	0.61
**PAAD**	5	66	0.97	2.0E-04	1.3E-03	37	0.75	0.94	0.74
**STAD**	7	78	0.89	3.5E-04	1.8E-03	37	0.50	0.73	0.59
**BLCA**	4	53	0.99	6.8E-04	2.9E-03	66	0.83	0.98	0.73
**PRAD**	4	105	0.91	2.4E-03	8.9E-03	21	0.48	0.81	0.56
**LUSC**	10	43	0.78	3.0E-03	9.6E-03	49	0.40	0.51	0.31
**HNSC**	4	80	0.87	6.6E-03	1.9E-02	30 and 39	0.39	0.55	0.11
**SARC**	6	79	0.76	1.8E-02	4.7E-02	39	0.27	0.57	0.40
**GBM**	3	94	0.72	9.6E-02	1.9E-01	10	-0.02	0.51	0.18
**UCEC**	14	61	0.60	1.3E-01	2.4E-01	62	0.34	0.56	0.48
**CESC**	6	64	0.63	1.5E-01	2.5E-01	27	0.24	0.32	-0.01
**SKCM**	3	55	0.68	1.5E-01	2.5E-01	35	0.24	0.43	0.10
**TGCT**	14	68	0.52	3.9E-01	4.9E-01	13	-0.01	0.14	-0.22
**THCA**	5	326	0.42	7.7E-01	8.3E-01	0 and 100	0.00	0.00	0.00
**COAD**	3	99	0.34	8.3E-01	8.3E-01	82	0.31	0.33	0.00

Supplementary Table 2: Predictivity of the HRDsum score for HRR status (class H1a BA/HM vs. class H3) and optimization of the cutpoints. The analysis was performed for cancer types with at least three tumors in the class H1a BA/HM. Optimal cutpoints for HRDsum refer to maximization of Youdens's index J = sensitivity + specificity - 1. Values for J are listed for the cutpoint 42, the optimal cutpoint and a leave-one-out cross-validation (loocv) analysis.

a

WES data

class	*N* (class)	cutpoint	sensitivity (%)	specificity (%)	PPV (%)	NPV (%)
**H1a, BA/HM**	93	42	100	40.2	43.3	100
**H1a**	97	42	97.9	40	44.2	97.6
**H1a + H1b**	129	42	87.6	39.3	52.6	80.5
**H1a + H2a**	99	42	98	40.4	45.1	97.6
**H1 + H2**	203	42	80.3	44.7	75.8	51.2
**H1a, BA/HM**	93	50	97.8	54.4	49.5	98.2
**H1a**	97	50	95.9	54.5	50.5	96.5
**H1a + H1b**	129	50	81.4	53	57.1	78.8
**H1a + H2a**	99	50	96	55.1	51.6	96.5
**H1 + H2**	203	50	73.4	62.8	81	52.2

b

Genotyping data (SNP arrays)

class	*N* (class)	cutpoint	sensitivity (%)	specificity (%)	PPV (%)	NPV (%)
**H1a, BA/HM**	93	42	98.9	47.1	46	99
**H1a**	97	42	97.9	47.5	47.5	97.9
**H1a + H1b**	129	42	88.4	48.8	57	84.5
**H1a + H2a**	99	42	98	48	48.5	97.9
**H1 + H2**	203	42	78.3	56.4	79.5	54.6
**H1a, BA/HM**	93	50	94.6	65.7	55.7	96.4
**H1a**	97	50	93.8	66.5	57.6	95.7
**H1a + H1b**	129	50	79.8	67.3	65.2	81.3
**H1a + H2a**	99	50	93.9	67.2	58.9	95.7
**H1 + H2**	203	50	63.5	69.1	81.6	46.8

Supplementary Table 3: Performance of HRDsum in the detection of HRR gene-altered ovarian carcinoma (TCGA-OV cohort). The classification task was to separate tumors in the listed class from tumors not in the listed class. **a** HRDsum calculated from WES data. **b** HRDsum calculated from genotyping data.

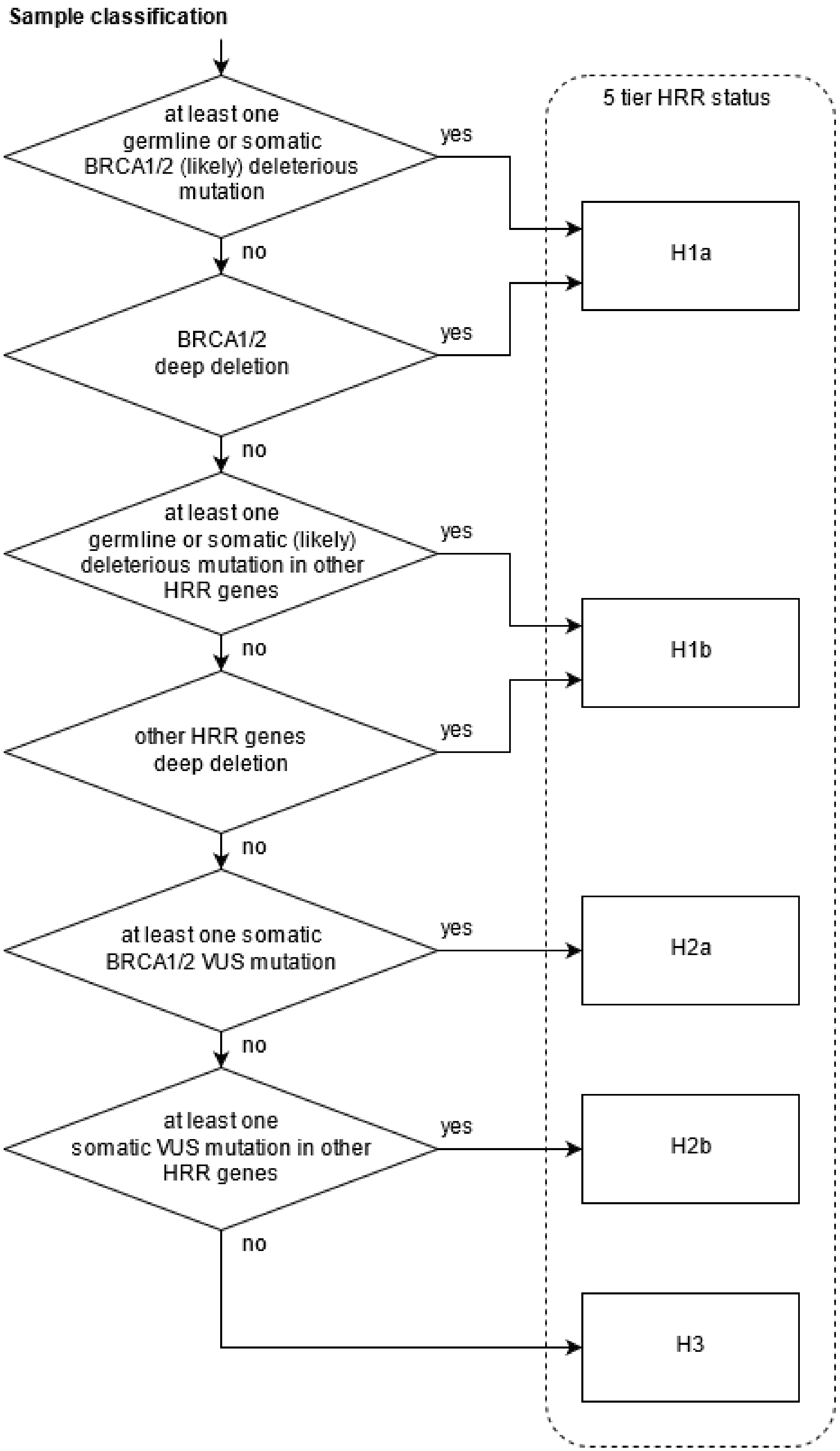

Supplementary Figure 1: Classification of HRR gene alterations resulting in a 5-tier classification system. Of a total of 8847 TCGA tumors 356 (4%) had deleterious *BRCA1/2* alterations (class H1a), 2277 (26%) deleterious alterations in other HRR genes (class H1b), 174 (2%) had VUS in *BRCA1/2* (class H2a) and 2267 (26%) had VUS in other HRR genes (class H2b).

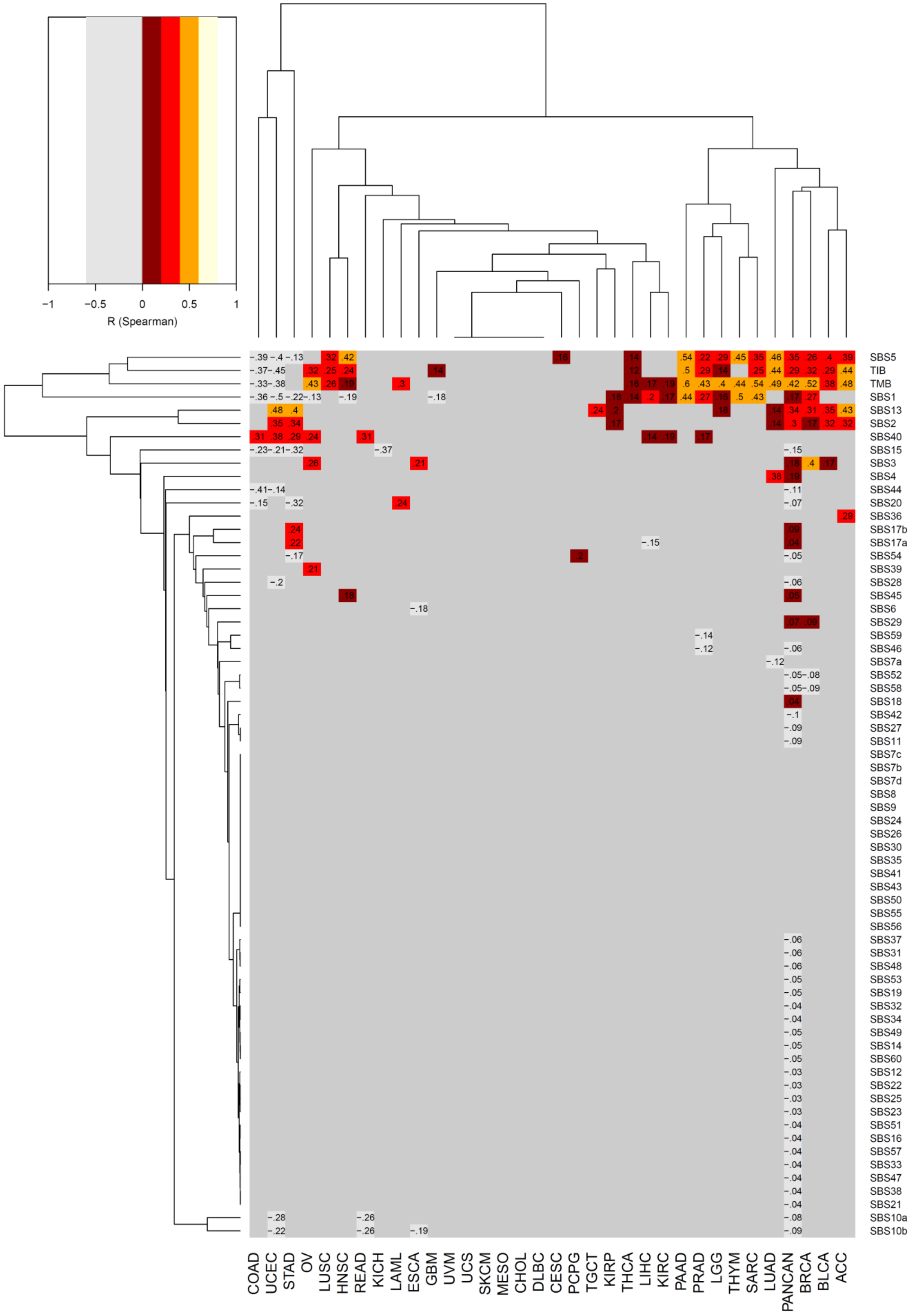

Supplementary Figure 2: Correlation analysis of TMB, TIB and the SBS mutational signatures with HRDsum pan-cancer and within specific cancer types. Significant positive correlations are shown in heat colors, while significant negative correlations are shown in light grey (FDR = 10%).

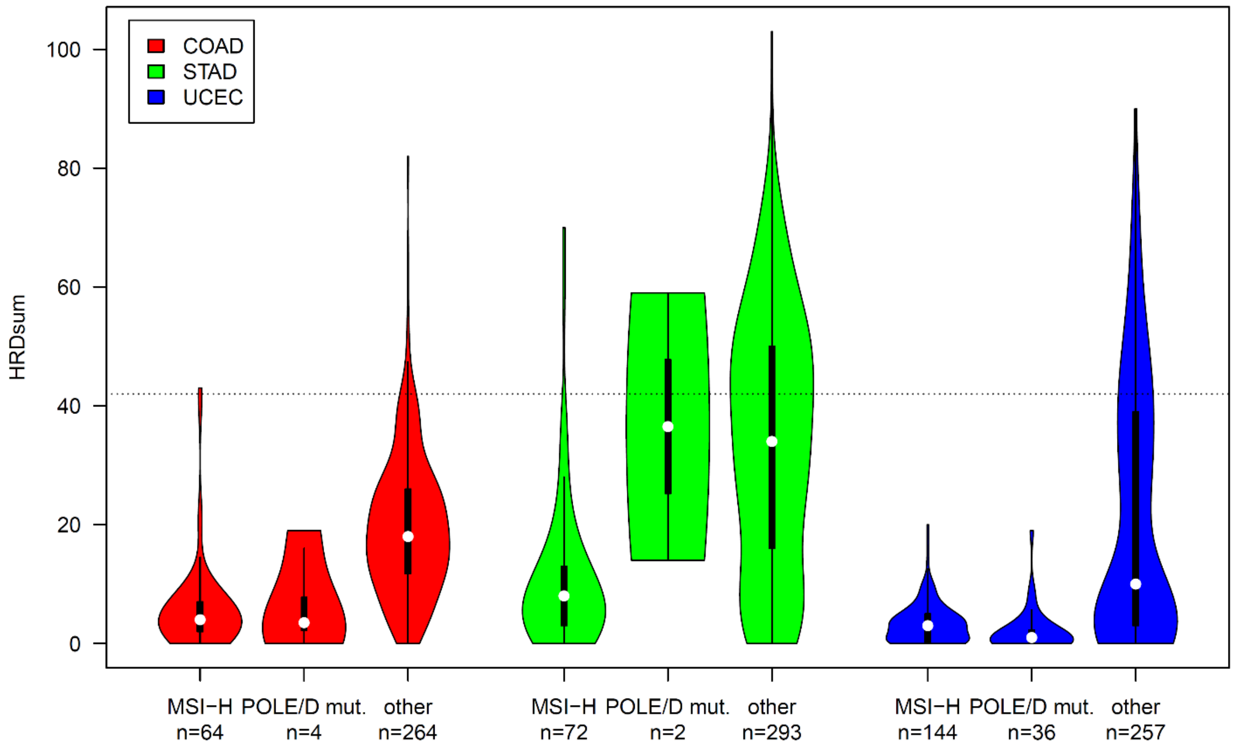

Supplementary Figure 3: HRDsum was lower in microsatellite-instable (MSI-H) and *POLE/D*-mutated tumors compared to tumors without these alterations in COAD, STAD and UCEC.

a

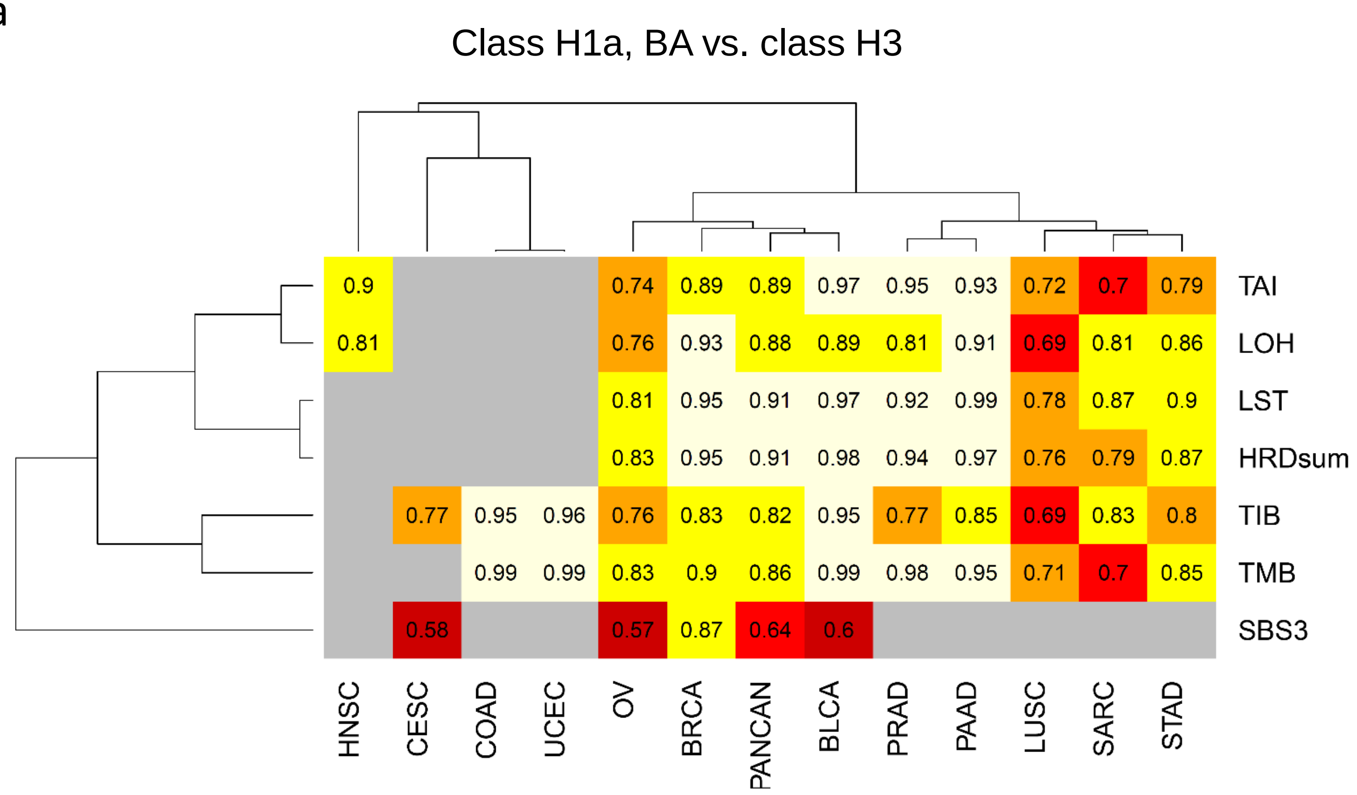

b

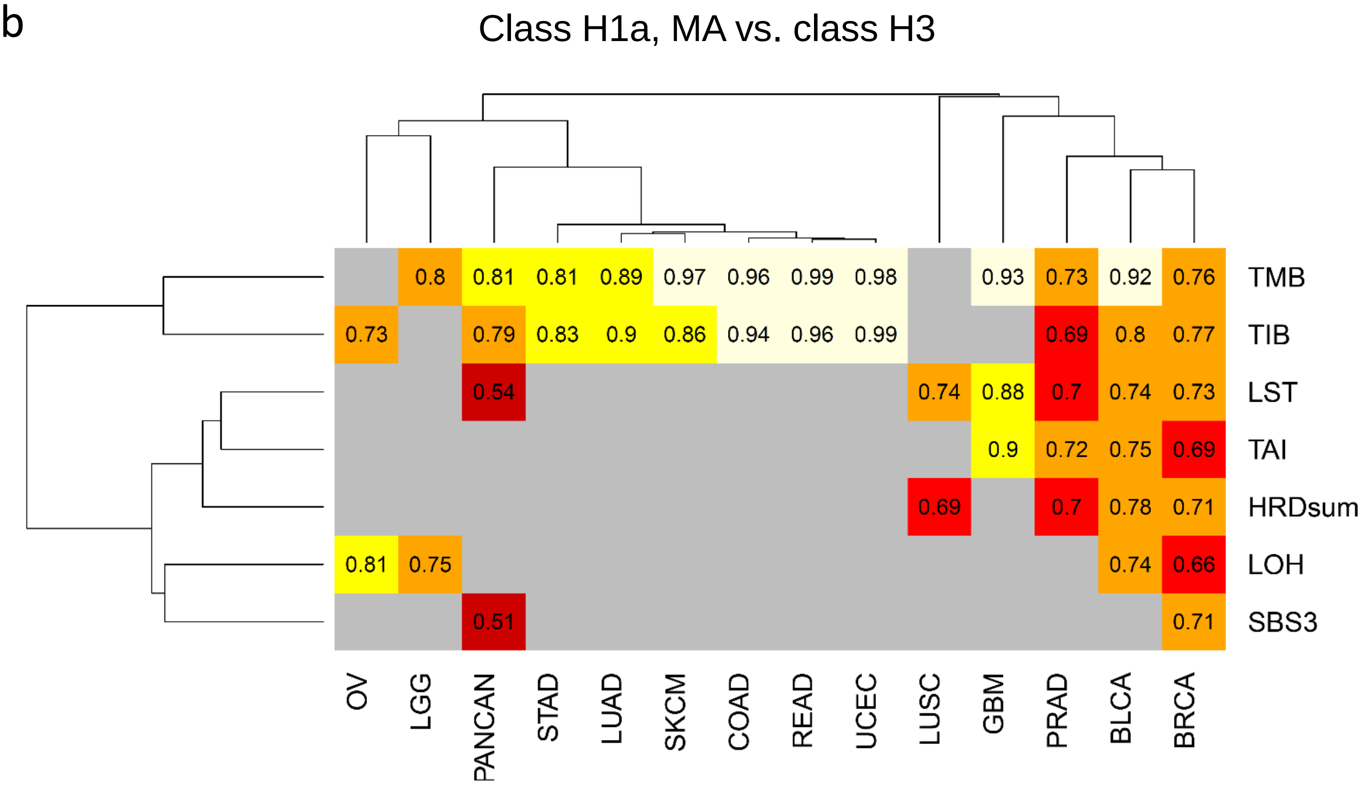

Supplementary Figure 4: Predictivity of HRDsum for biallelic (BA) and for monoallelic (MA) *BRCA1/2* alterations. **a** ROC analysis of tumors with biallelic *BRCA1/2* alterations (class H1a, BA) *versus* tumors with unaffected HRR genes (class H3). **b** ROC analysis of tumors with monoallelic *BRCA1/2* alterations (class H1a, MA) *versus* tumors with unaffected HRR genes (class H3).

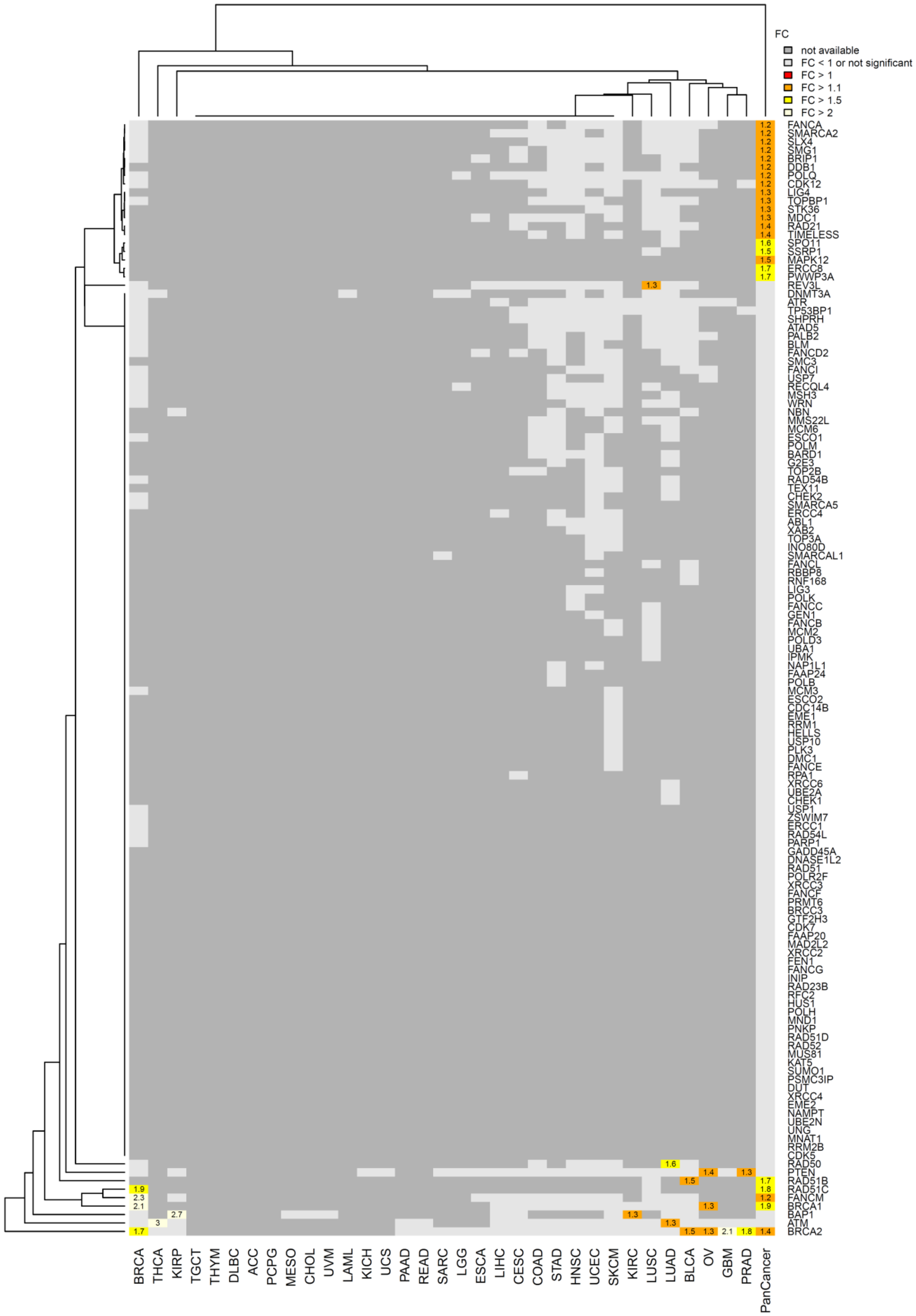

Supplementary Figure 5: Correlation of the level of HRDsum with alterations in *BRCA1*, *BRCA2* and 140 other HRR genes. For each of the genes, tumors with deleterious alterations or VUS were compared to tumors without mutation and without *BRCA1*-hypermethylation. FC = Fold change between mutated and wildtype tumors. Light grey: Non-significant result. Dark grey: not analyzed (less than five mutated tumors available).

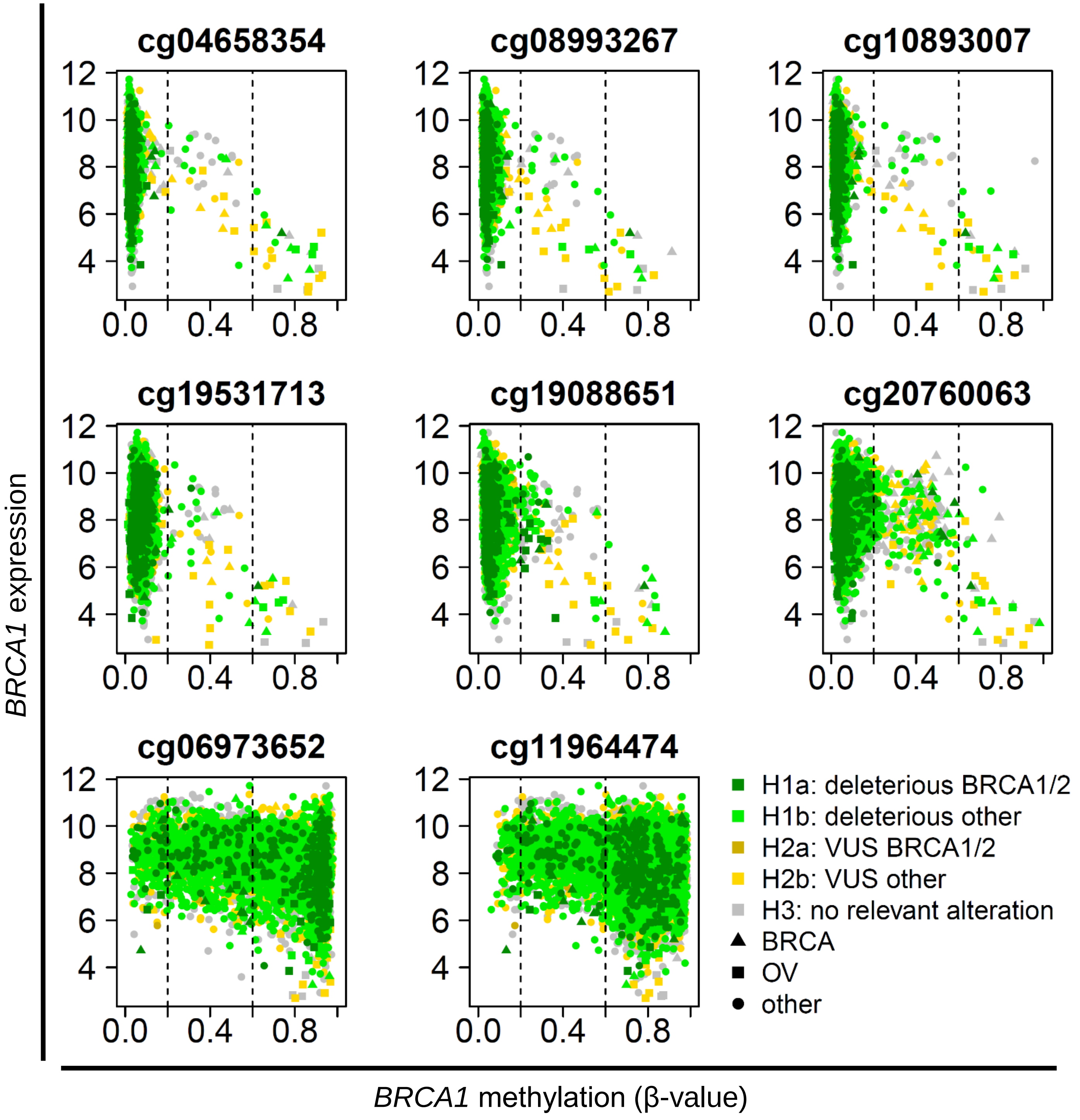

Supplementary Figure 6: Correlation of *BRCA1* expression (log2 scale) and *BRCA1* methylation (beta-values). Tumors were classified as strongly hypermethylated if $\beta \geq 0.6$ and as moderately hypermethylated if $\beta \geq 0.2$ for at least one of the CpG sites cg04658354, cg08993267, cg10893007 and cg19531713.

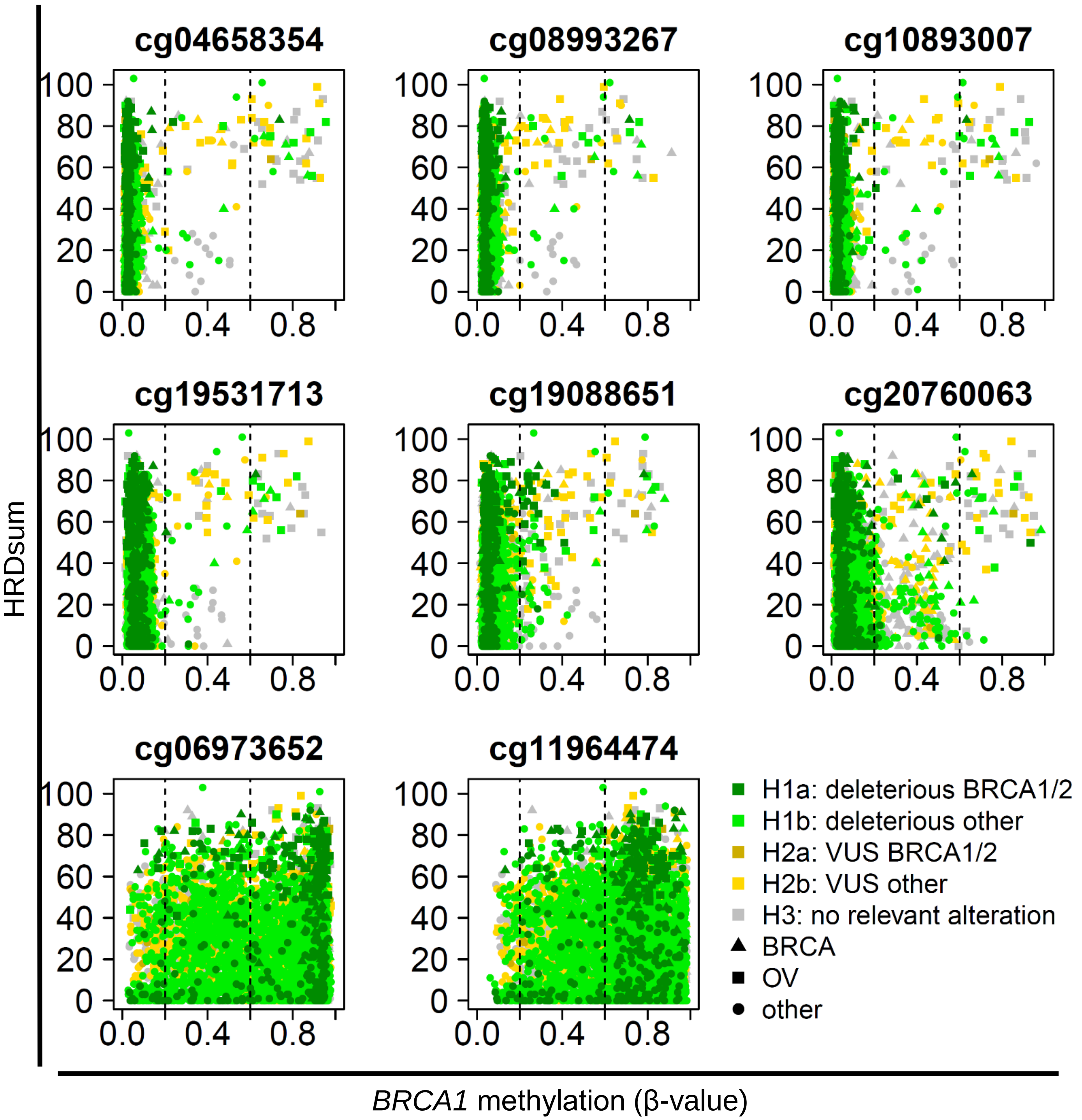

Supplementary Figure 7: Correlation of HRDsum and *BRCA1* methylation (beta-values). Tumors were classified as strongly hypermethylated if β ≥ 0.6 and as moderately hypermethylated if β ≥ 0.2 for at least one of the CpG sites cg04658354, cg08993267, cg10893007 and cg19531713.

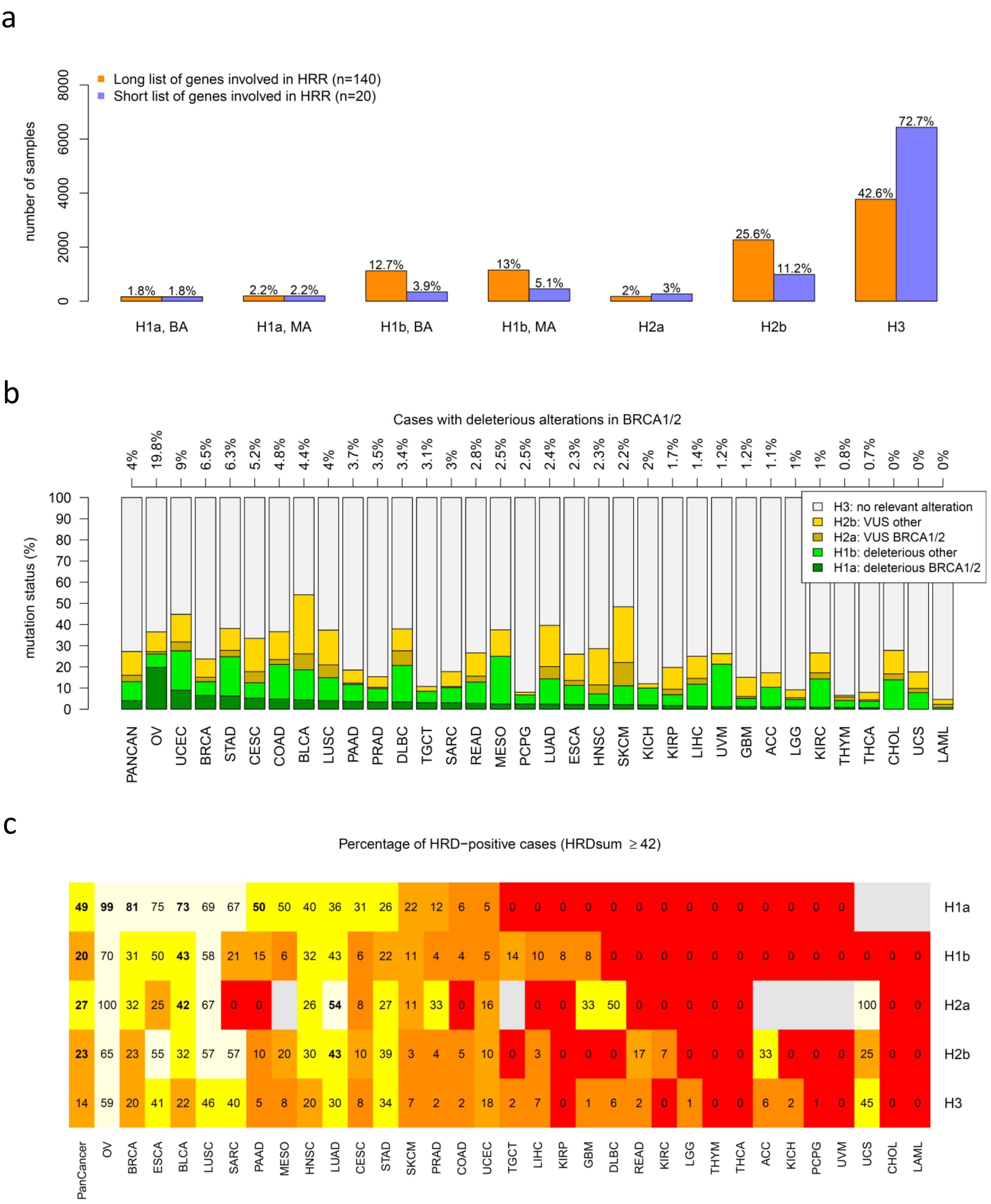

Supplementary Figure 8: Using a short list (n=20) instead of a long list (n=140) of HRR genes for tumor classification. **a** Numbers of tumors in the classes H1-H3 across all cancer types. MA = monoallelic alteration, BA = biallelic alteration. **b** Tumors with deleterious alterations in *BRAC1/2* (H1a), in other HHR genes (H1b), VUS in *BRCA1/2* (H2a) and in other HRR genes (H2b). Same as Fig. 1 A, but for the short gene list. **c** Percentage of HRD-positive (HRDsum ≥ 42) tumors in the five classes H1a-H3 (grey = no cases). Significant enrichments of HRD-positive tumors compared to H3 are shown in bold face. Same as Fig. 1C, but for the short gene list.

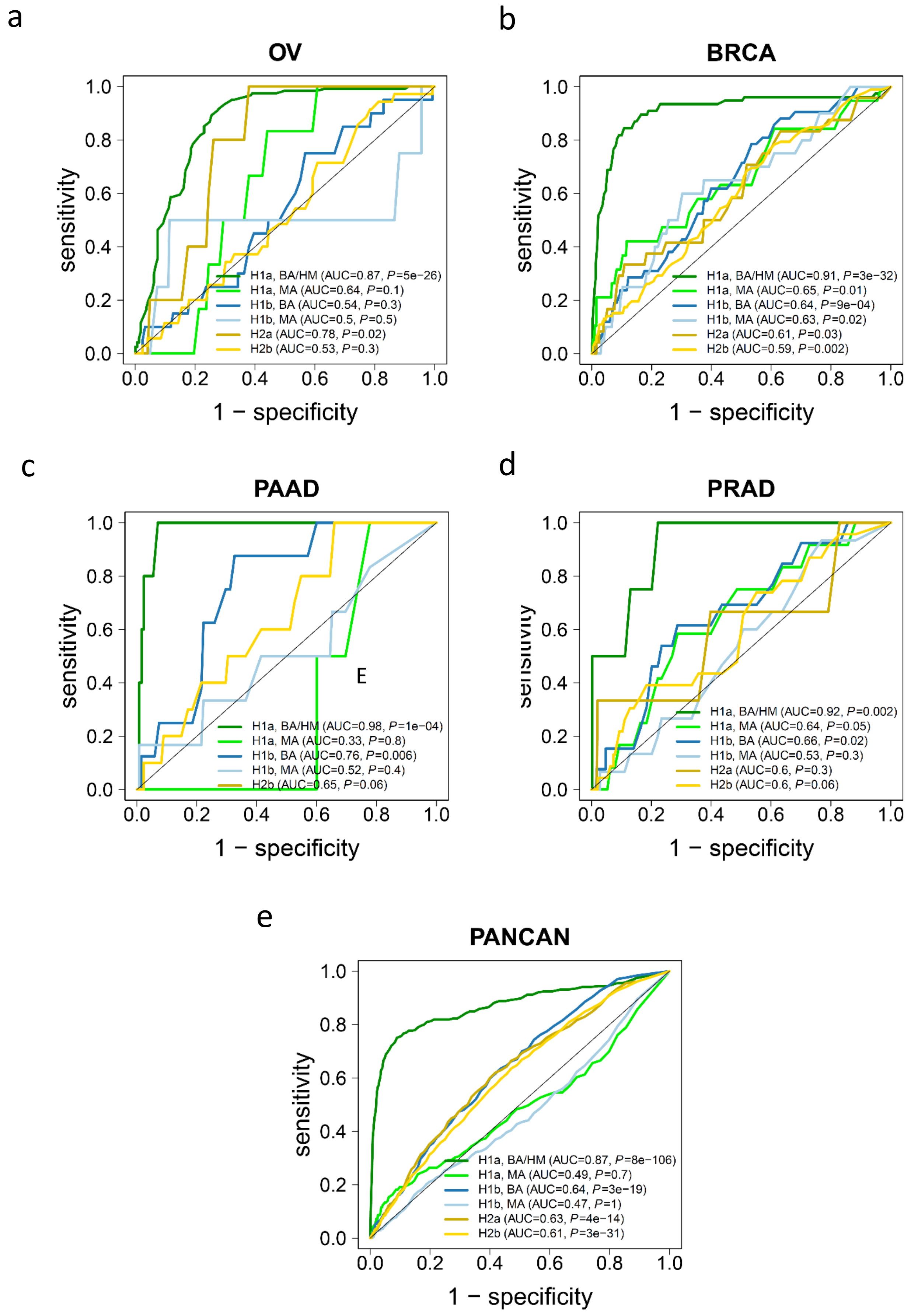

Supplement Figure 9: Separation of tumors with alterations in HRR genes (classes H1a-H2b) from tumors without such alterations (class H3) by HRDsum. Same as Fig. 5, but the short list (n=20) of HRR genes was used for tumor classification. **a** Ovarian cancer (OV). **b** Breast cancer (BRCA). **c** Pancreatic adenocarcinoma (PAAD). **d** Prostate adenocarcinoma (PRAD). **e** Across 33 cancer types (PANCAN).

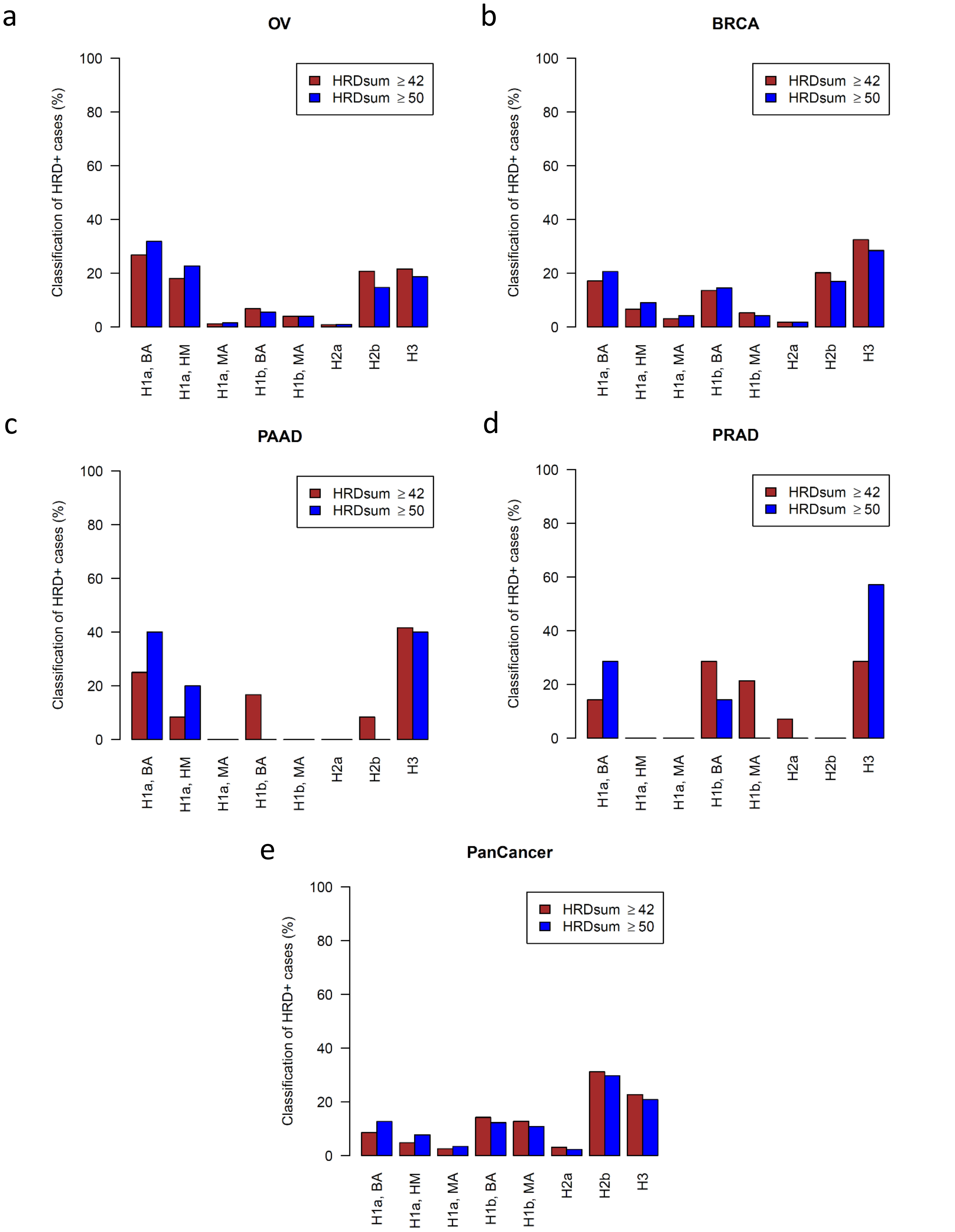

Supplementary Figure 10: Explanation of high HRDsum scores (HRDsum ≥ 42) by genetic and epigenetic alterations in HRR genes. **a** Ovarian cancer (OV). **b** Breast cancer (BRCA). **c** Pancreatic adenocarcinoma (PAAD). **d** Prostate adenocarcinoma (PRAD). **e** Across 33 cancer types (PanCancer).

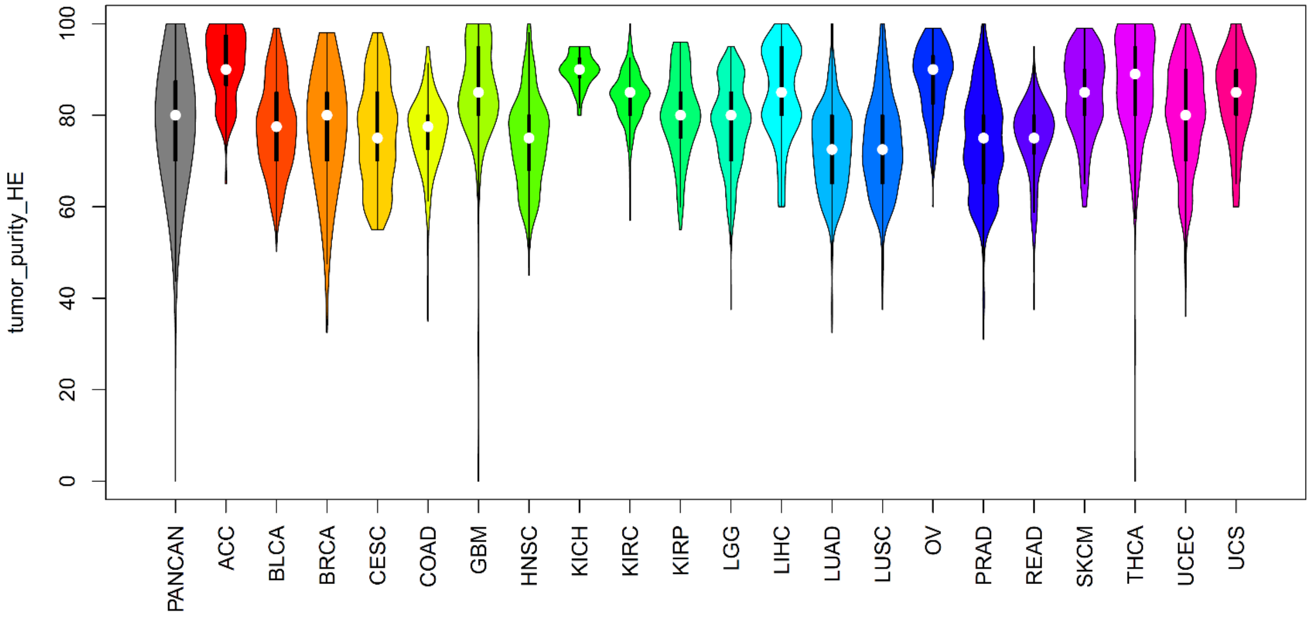

Supplementary Figure 11: Tumor purity in the TCGA cohort. Tumor cell content determined by pathologists from HE-stained slides. It was planned to include only samples with minimum tumor content 80% in the TCGA cohort.

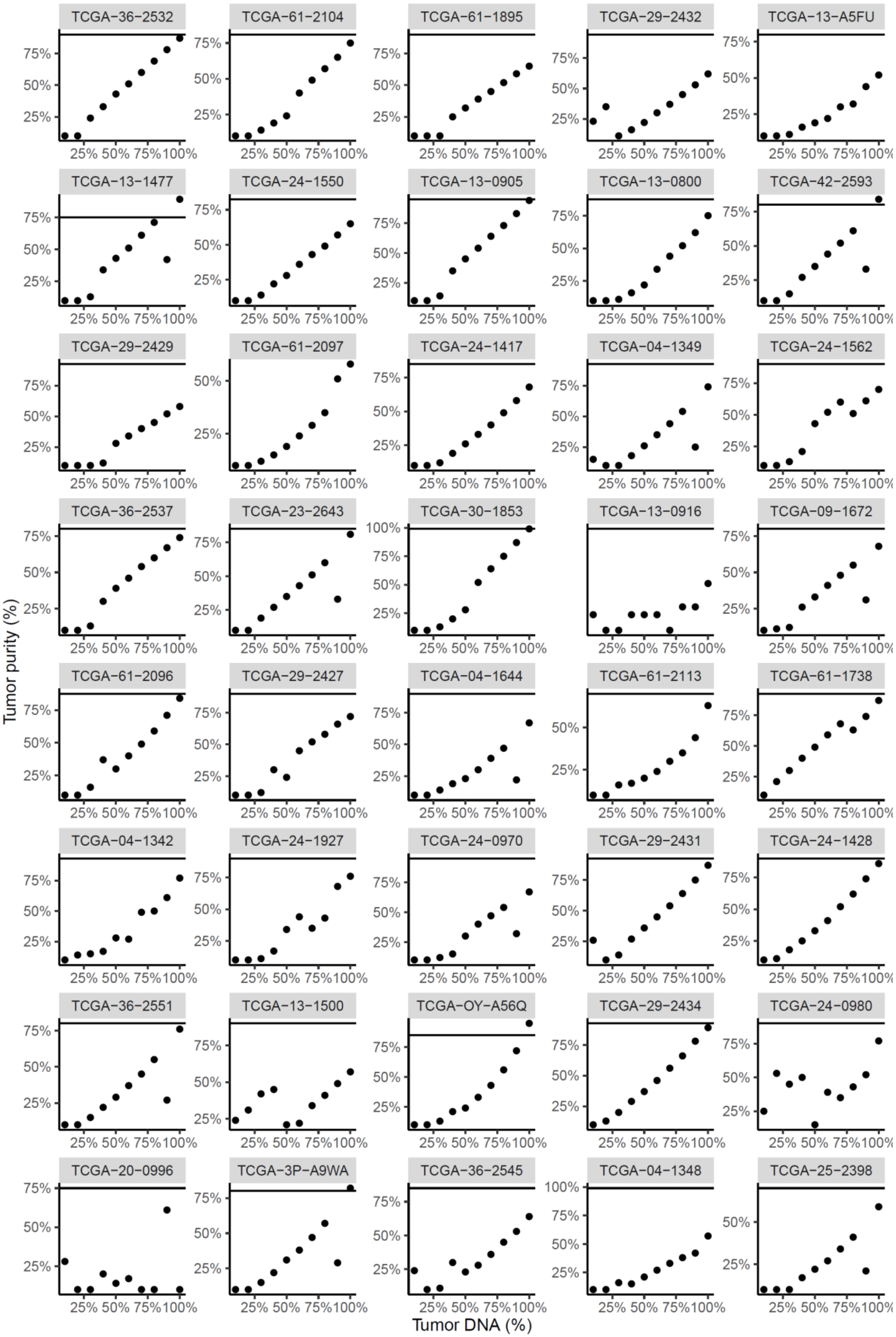

Supplementary Figure 12: *In silico dilution* experiment including 10%, …, 100% of reads from tumor DNA samples and 90%, …, 0% of reads from the corresponding normal DNA samples. Correlation of tumor purity (estimated by Sequenza) with the level of dilution.

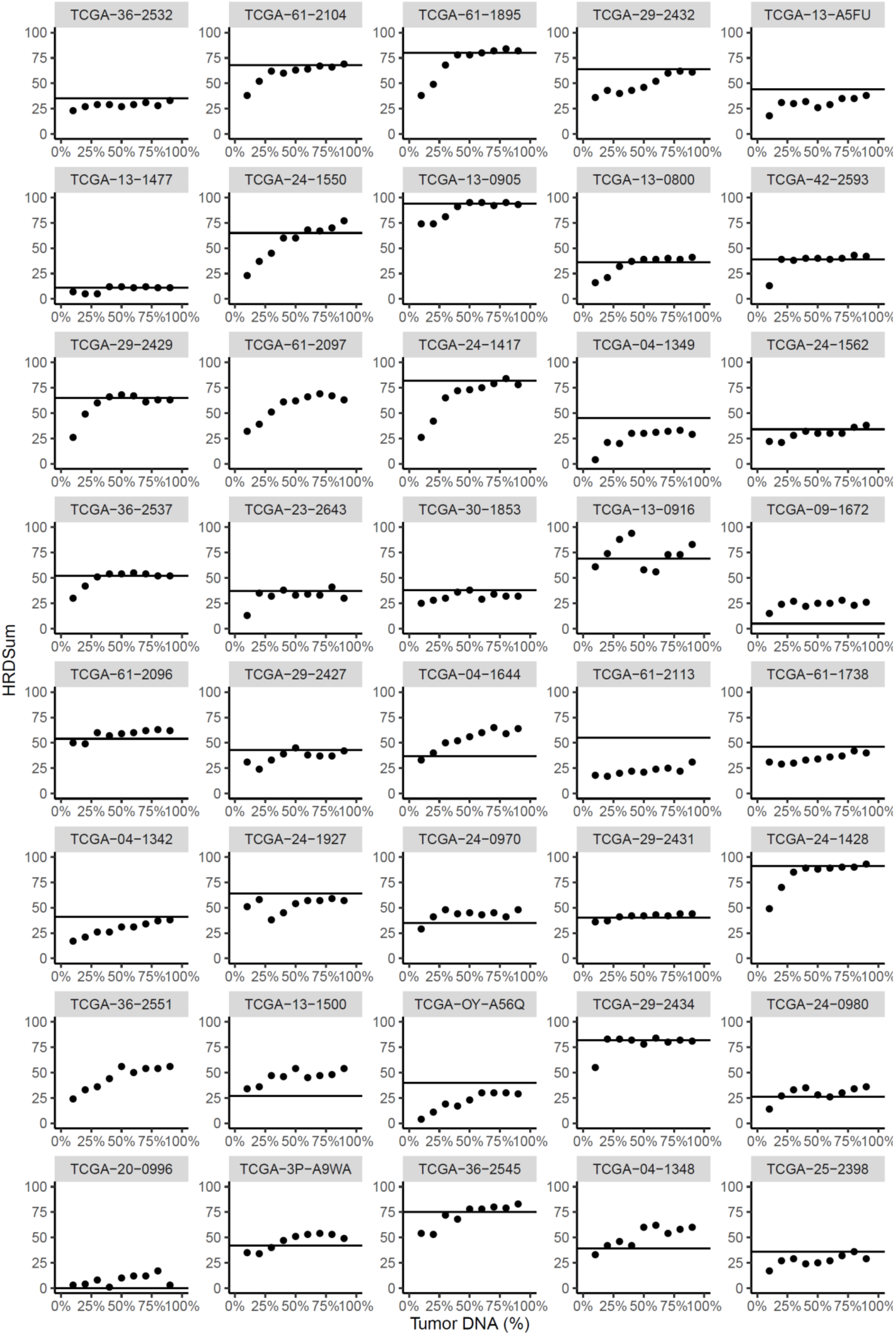

Supplementary Figure 13: *In silico* dilution experiment including 10%, …, 100% of reads from tumor DNA samples and 90%, …, 0% of reads from the corresponding normal DNA samples. HRDsum scores calculated from WES data (dilution series, points) and from genotyping data (undiluted samples, line).
